# Blood Transfusion and Spread of Variant Creutzfeldt-Jakob Disease

**DOI:** 10.3201/eid1301.060396

**Published:** 2007-01

**Authors:** Klaus Dietz, Günter Raddatz, Jonathan Wallis, Norbert Müller, Inga Zerr, Hans-Peter Duerr, Hans Lefèvre, Erhard Seifried, Johannes Löwer

**Affiliations:** * University of Tübingen, Tübingen, Germany; † Freeman Hospital, Newcastle upon Tyne, United Kingdom; ‡ University Hospital Essen, Essen, Germany; § University of Göttingen, Göttingen, Germany; ¶ DRK Blood Donor Service West, Hagen, Germany; # DRK Blood Donor Service Baden-Württemberg, Hessen, Frankfurt am Main, Germany; ** Paul-Ehrlich-Institute, Langen, Germany

**Keywords:** blood transfusion, blood donors, variant Creutzfeldt-Jakob disease, models, theoretical, biometry, infection, endemic diseases, epidemiology, communicable diseases, emerging, risk assessment, research

## Abstract

Variant Creutzfeldt-Jakob disease (vCJD) may be transmissible by blood. To prevent secondary transmission through blood components, several countries have started to exclude as donors persons who have received a blood transfusion. We investigated the effectiveness of this measure by using a dynamic age-structured model. It is the first such model based on epidemiologic data: 1) blood donor activities, 2) a case-control study on CJD, 3) age distribution of recipients, and 4) death of recipients of blood transfusions. The model predicts that an infection like vCJD, which has been introduced into the population by the alimentary route, could not become endemic by transfusion alone and that <1% of cases would be avoided by excluding from blood donation those persons who have received a transfusion.

## Abstract 2

The effect of reducing vCJD transmission by excluding potential blood donors who have received a blood transfusion can be quantified and depends on the absolute number of cases observed or expected.

Recent studies of variant Creutzfeldt-Jakob disease (vCJD) indicate that this disease is transmissible by blood. One case of probable transfusion-transmitted vCJD infection has been reported, and 1 case of subclinical infection has been detected (*1*,*2*). On February 9, 2006, a third case was announced by the UK Health Protection Agency (http://www.hpa.org.uk/hpa/news/articles/press_releases/2006/060209_cjd.htm). Each of the 3 patients had received a blood transfusion from a donor who subsequently developed clinical vCJD, which indicates that transfusion caused the infection. However, a policy to exclude potential donors who had received a transfusion would not have prevented at least the first 2 cases because the corresponding donors had not received any blood transfusion. Diagnostic tools to detect prions in blood are under development (*3*), but no routine test for the presence of the infectious agents of vCJD is available. Therefore, the questions arise as to whether an infection like vCJD could become endemic through blood donation alone and to what extent exclusion of potential donors with a history of transfusion would influence the transmission of such an infection (i.e., how many deaths due to the infection could be prevented?). The following mathematical model is the first to address these questions on the basis of epidemiologic data and realistic and epidemiologically justified assumptions.

## Methods

### Model Structure

**Figure 1:**
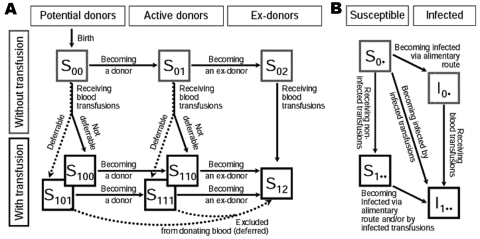
A) States and transitions for the model of blood transfusion in the absence of an infection. B) Routes of infection. The arrows representing deaths out of all states are omitted. Paths of donor exclusion are plotted by dotted arrows. S_00_, nonrecipients who do not donate; S_01_, nonrecipients who donate; S_02_,nonrecipients who are excluded from donating; S_100_,recipients who do not donate; S_101_, recipients who become excluded from donating; S_110_, recipients who donate; S_111_, recipients who become excluded from donating; S_12_, recipients who are excluded from donating. Indices replaced by a dot (panel B) represent all other possible states (e.g., S_0•_ represents S_00_, S_01_, or S_02_).

Figure 1A shows the transitions of a person through the basic states of potential donor activities and receipt of blood transfusion. After birth a person is in the state of not having received any transfusion and not yet being an active donor (S_00_). The first index refers to the person’s state as a transfusion recipient; the second index, to the person’s status as a donor. Persons in state S_00_ can change to state S_01_ by becoming a donor or to state S_100_ or S_101_ by receiving a blood transfusion. The third index indicates whether a person with a transfusion history can actually be identified and excluded from donating blood (deferred) (index 1) or not (index 0). The states S_111_ and S_110_ can be reached by either transfusion recipients who start donating blood or active donors who receive a blood transfusion. Blood donors who become inactive are transferred into the states of ex-donors S_02_ and S_12_, depending on their transfusion history. Ex-donors can also become transfusion recipients; i.e., they are transferred from S_02_ to S_12_. Donor exclusion transfers a certain proportion of transfusion recipients into the state of ex-donors. For all susceptible states, Figure 1B shows the transitions to the corresponding infected states. Table 1 provides a list of all input parameters together with descriptions and sources. The details of the model with all the numerical parameter estimates and the equations are given in the Technical Appendix . The computer program is available upon request. This article summarizes the major features of the model, the data sources, and the estimation of the model parameters.

**Table 1:** Summary of input parameters for the model*

Parameters	Description	Source
Age-specific mortality rates	U-shaped, with minimum at age 10.	Federal Statistical Office of Germany
Donor recruitment	Donors ages 18–67 y. Maximum recruitment rate at age 18, lower plateau ages 25–50; further decrease until age 67.	Age-distribution of first-time donors at DRK Blood Service and age structure in population
Proportion of donors	3% of population.	DRK Blood Service West
Duration as active donor	Donors ages 18–40 y, mean duration as active donor 10–14 y, decreases linearly to 0.	Age distribution of active donors at DRK Blood Service West, by age at first donation
Risk of receiving transfusions	Bimodal, with peaks for newborns and aged persons. Multiple transfusions possible.	Data collected from 4,867 patients March 2003, University Hospital Essen, Germany
Transfusion-associated risk for death	Increases according to a sigmoid function, ≈17% at birth to ≈48% in old age. For those with transfusion-associated risk for death, life expectancy is ≈2.5 years at birth and decreases to ≈0.5 y in old age.	Follow-up of ≈3,000 transfusion recipients for ≈7.5 y in Newcastle, UK (*4*)
Alimentary infection	Constant over an initial period of 10 y.	Arbitrary assumption
Incubation period†	Gamma distributed with mean 16 y, SD 4 y. Sensitivity analysis with mean = 50 y and same coefficient of variation.	Models fitted to the UK incidence of vCJD (*5*,*6*)
Donor exclusion	Either 0 or 95% of those with transfusion history.	Arbitrary assumption

*DRK, German Red Cross; SD, standard deviation; vCJD, variant Creutzfeldt-Jakob disease. †Time between infection and death, i.e., duration of infection.

### Demography

To simplify the model, we did not attempt to describe the demographics of the population during the next 150 years. Doing so would involve predicting changes in rates of birth, death, and immigration. It is assumed that in the absence of infection, the population is demographically stationary. We assumed a constant inflow of newborns and an age-specific death rate. The latter was estimated as a weighted mean of the age-specific female and male death rates. Because this study was initiated in Germany, we used the corresponding demographic data. To start the simulation in a demographically stationary state, the model was run for 100 years without infection. Thus, the age distribution of the population was identical to the life table of Germany 2002/2004 averaged over both sexes (http://www.destatis.de/download/d/bevoe/sterbet04.xls).

### Modeling Blood Donors

Blood donors in Germany are >18 and <68 years of age. The rates for becoming a new donor and terminating the period as an active donor are age dependent. The corresponding parameters were estimated by using data from 262,071 donors registered with the German Red Cross (DRK) Blood Service West in Hagen, Germany, including age, sex, age at first donation, number of donations, and date of last donation.

The age-specific prevalence of active donors peaks at ≈24 years of age and subsequently declines monotonically to zero by age 68. The overall prevalence in the population is 3%, i.e., 2.4 million donors in a population of ≈80 million.

### Modeling Transfusion Recipients

The model takes into account that persons may receive >1 transfusion throughout their lifetime, but it does not track the number of transfusions received per person. Persons with >1 transfusion continue to be at risk for infection from further transfusions. The age-specific risk of receiving a transfusion was estimated from data for all patients hospitalized at the University Hospital in Essen during March 2003. Of 4,867 patients, 1,343 (27.6%) received >1 transfusion. The number of persons receiving a blood transfusion in each 5-year age group was divided by the corresponding number of persons in the general population. The observed rates were fitted with a simple model that assumes initially an exponential decline and subsequently a unimodal peak, which is proportional to the density function of the normal distribution. These age-specific ratios were properly scaled to balance the yearly number of transfusions per capita. To limit the complexity of the model, we did not take into account persons in subgroups, such as those with hemophilia, who obtain blood products from pools of donors. Because for medical reasons these subgroups are excluded from donating blood, they cannot contribute to persistence of the infection.

### Independence of Receiving and Donating Blood

The events of receiving a blood transfusion and of donating blood are assumed to be independent of each other. This assumption is supported by the results of a case-control study of potential risk factors for CJD, which was coordinated by the Clinical Surveillance Centre for CJD, Department of Neurology in Göttingen, Germany (*7*). Table 2 shows the joint distribution for the control group of having received and donated blood. According to the Fisher exact test, the p value for the hypothesis of no association is 0.43.

**Table 2:** Joint distribution of transfusion history and blood donation

Received blood	Donated blood, no. observed (no. expected if events are independent)		Total no. (%)
	No	Yes	
No	401 (404)	104 (101)	505 (82)
Yes	93 (90)	19 (22)	112 (18)
Total no. (%)	494 (80)	123 (20)	617 (100)

Heterogeneity in the risk of receiving a blood transfusion is modeled by the assumption that only a proportion of the population are at risk, whereas the remaining proportion never receives a transfusion. This assumption was introduced to be consistent with data from the case-control study, in which ≈18% of the population reported having ever received a blood transfusion. Without this assumption, the model would predict that eventually 100% of a cohort would receive a blood transfusion because the average annual risk of receiving a blood transfusion is about 5%, i.e., ≈4 million in a population of 80 million.

### Modeling Transfusion-associated Death Rates

The transfusion-associated death rate has been described in detail by Wallis et al. (*4*). A good fit to the data assumes that at all ages a certain proportion of transfusion recipients have a higher rate of dying and the remaining proportion has a survival rate that corresponds to that of persons of the same age group in the general population. This age-dependent proportion of transfusion recipients with an increased risk for death is described by a generalized logistic function with a positive value at birth and an asymptote <100% for old age. The transfusion-associated death rate increases linearly with age. The increased death rate appears to be concentrated in the first 2 years after a transfusion. Wallis et al. report that 2,888 patients were observed as long as 7.4 years after transfusions received in June 1994 (*4*). The sex-specific rates were averaged for the simulation model.

### Modeling the Infection

Usually the incubation period refers to the time between the infection and disease. In the context of CJD, however, disease can refer to onset, diagnosis, or death. Like Bacchetti, we also focused on death rates (*8*–*10*). The incubation period is assumed to be γ distributed with a mean duration of 16 years and a standard deviation of 4 years, which conforms to estimates of Valleron et al. and Ghani et al. (*5*,*6*). Because of great uncertainty about the length of the incubation time, we also considered a much higher value of 50 years in the absence of the competing risk for death. The coefficient of variation is assumed to be the same, such that the standard deviation is 12.5 years. Because of competing risks, the actual sojourn in the incubation period is 15.3 for an incubation period of 16 years and 34.0 years for an incubation period of 50 years. The proportions of infected persons who would die with disease symptoms are 79% and 37% for the incubation periods of 16 and 50 years, respectively. This means that for an incubation time of 50 years, nearly two thirds would die without disease symptoms. Hereafter we refer to these values of 15 and 50 years as short and long incubation periods.

We distinguish between 2 modes of transmission. Initially, the infection is introduced into the population by the alimentary route. In the United Kingdom the number of infected animals entering the food supply peaked in 1989; most were concentrated within a period of 10 years (*11*), which we take as the assumed period of alimentary infection. After this period, this mode of transmission was interrupted so that further transmissions are possible only through blood transfusions.

A study to detect the presence of abnormal prion protein in appendix and tonsil tissues has suggested a prevalence of 235 infections per million in the United Kingdom (*12*). We arbitrarily assumed the prevalence of infections in Germany to be ≈1 order of magnitude lower, yielding a cumulative incidence of 25 per million, which was the value used for the simulations.

We made 2 contrasting assumptions about the infectivity of blood preparations and evaluated the results of these 2 simulations: each transfusion (100% infectivity) or no blood transfusion (0% infectivity) from an infected donor leads to infection of the recipient. In the model the infection probability (probability of receiving blood from an infected donor) is proportional to the proportion of infected donors among all donors. Thus, we can calculate the number of infections from blood transfusions compared with the number of infections from alimentary transmission alone.

### Modeling Donor Exclusion

The model distinguishes between persons with and without transfusion history, termed recipients and nonrecipients; these terms are applied to persons whether they have or have not donated blood. The model allows recipients to be excluded from donating blood. In modeling the exclusion of recipients, we took into account that this measure may be imperfect and that a certain proportion of recipients may not be excluded.

## Results

**Figure 2:**
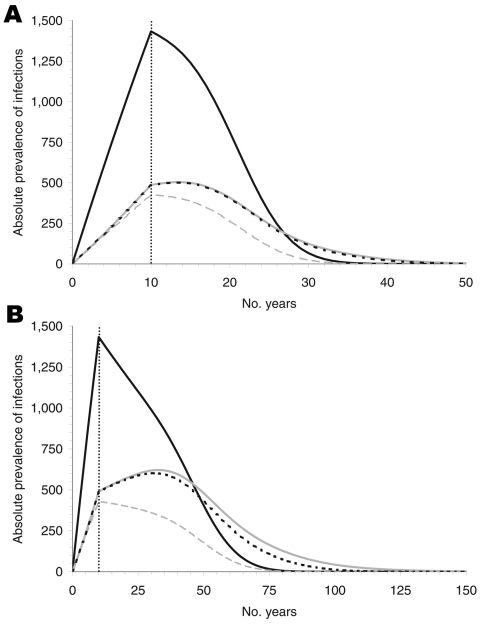
Absolute prevalence of infection for an incubation period of 16 (A) and 50 (B) years, for nonrecipients of blood transfusion (solid, black), recipients under the assumption of no infectivity (dashed, grey), of 100% infectivity without donor exclusion (dotted, black), and 100% infectivity with donor exclusion (solid, gray). The prevalence declines after the alimentary route of transmission is interrupted, i.e., after 10 years. Prevalence differs only slightly if the infection probability of a transfusion from an infected donor is increased from 0% to 100%. Donor exclusion produces negligible reductions.

For the parameter estimates obtained from the sources described above, the infection cannot become endemic (Figure 2). If we assume no further spread through blood transfusions after 10 years of infections by the alimentary route, the maximum prevalence reached is ≈1,860 (1,434 for nonrecipients plus 426 for recipients) because some of the infected persons die of other causes during the incubation period. If transmission is assumed to be possible through blood transfusions (100% infectivity), then the maximum prevalence among recipients is increased by ≈78 infections after 4 more years for the short incubation period and by 193 infections after 23 more years for the long incubation period.

We assumed that donor exclusion is implemented immediately at the beginning of the alimentary infection risk period, which reduced the original number of 2.55 million donors by ≈20% to a value of 2.05 million donors. Because the model does not account for the stock of blood donations, this reduction in the number of donors must be compensated for with an increased rate of donations per donor to satisfy the demand; i.e., the average number of donations would have to increase from 1.6 to 2 per donor per year. Figure 2A shows that donor exclusion has almost no effect when the incubation period is assumed to be 16 years. The absolute prevalence (i.e., the actual number of infected persons) differs at most by 9. For a long incubation, differences are visible (59 persons at most) but small in view of the long time intervals and the size of the total population (Figure 2B). The reason for these small differences is described below.

The cumulative numbers of deaths from the infection are given in Table 3. The numbers are considerably smaller for the long than for the short incubation period because a long incubation period implies more deaths from other causes. The numbers are given separately for cases in patients with and without a history of blood transfusion. The route of infection for nonrecipients is alimentary only, whereas the route of infection for recipients is unclear. If we compare the simulations at 100% and 0% infectivity of blood transfusions, we observe 172 and 224 additional cases for the short and the long incubation periods, respectively. These numbers represent 11% of 1,557 and 31% of 725 cases, which would be expected for 0% infectivity for the short and long incubations periods, respectively. For the short incubation period we expect a higher absolute number of alimentary cases but a smaller proportion of transfusion cases than for the long incubation period. The exclusion of donors would prevent only 15 and 50 cases, i.e., ≈15 (0.9%) of 1,729 and 50 (5%) of 949, respectively, at the end of the epidemic. The epidemic lasts for ≈50 or ≈150 years for the short and the long incubation periods, respectively.

**Table 3:** Cumulative numbers of deaths from variant Creutzfeldt-Jakob disease at the end of the epidemic

Incubation period	Donors excluded	Infectivity (%)	Without transfusion	With transfusion	Total no. cases
Short	No	0	1,167	390	1,557
	No	100	1,167	562	1,729
	Yes	100	1,167	547	1,714
Long	No	0	503	222	725
	No	100	503	446	949
	Yes	100	503	396	899

**Figure 3:**
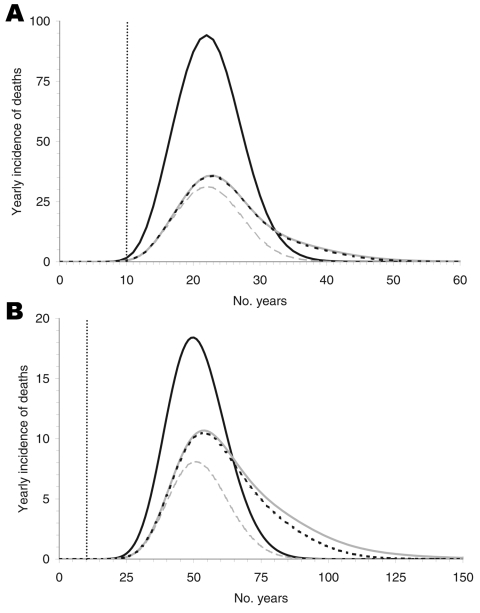
The yearly incidence of deaths for an incubation period of 16 (A) and 50 (B) years. The black curves show nonrecipients of blood transfusion who were infected only by the alimentary route. These curves are independent of the infection probability and the rate of donor exclusion. The lower 3 curves represent the deaths of recipients originating from 0% infectivity of blood transfusions (dashed gray), 100% infectivity without donor exclusion (solid gray), and 100% infectivity of blood transfusions with donor exclusion (dotted black, almost indistinguishable from solid gray line in A). The differences between the solid and dashed gray curves represent the cases due to blood transfusion.

The predicted yearly incidence of deaths due to vCJD, separated by transfusion history, is shown in Figure 3. The yearly peak incidence of total deaths would be 128 and 29 for the short and the long incubation periods at 23 and 51 years after the beginning of the epidemic, respectively. For 0% infectivity the peak incidence would be only 5 and 3 cases less for the short and long incubation periods, respectively, which implies that the exclusion of donors with a transfusion history does not effectively prevent infection.

**Figure 4:**
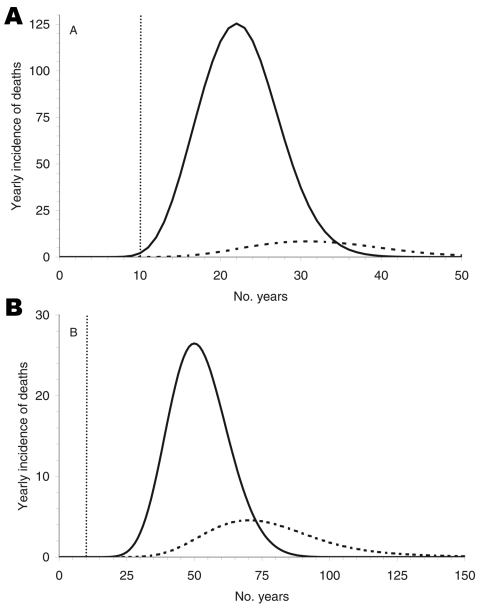
Yearly incidence of deaths caused by alimentary transmission (solid line) and by blood transfusion (dashed line).The 2 peaks differ by 9 and 20 years, depending on the incubation period: 16 (A) and 50 (B) years, respectively.

Figure 4 shows the predicted yearly incidence of deaths according to the route of infection. The time lags between the peaks of deaths due to alimentary infection and due to transfusion clearly differ and are 9 and 20 years for short and long incubation periods, respectively.

**Figure 5:**
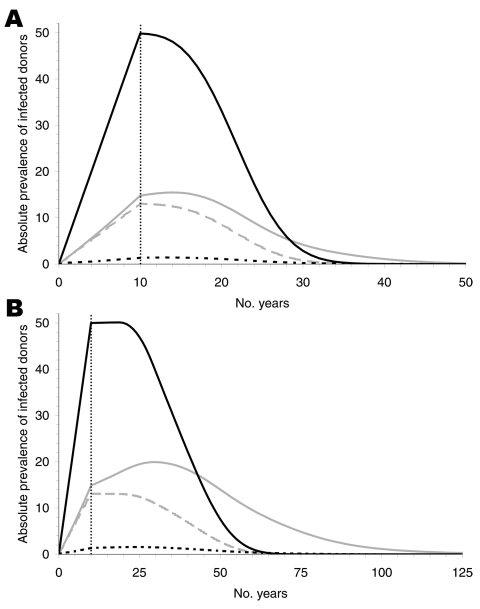
Absolute prevalence of infected donors for an incubation period of 16 (A) and 50 (B) years. The solid black curves show the infected donors without transfusion history. These curves are identical for 0% and 100% infectivity and are independent of donor exclusion. The gray curves show infected donors with transfusion history for 100% (solid) and 0% (dashed) infectivity, respectively, without donor exclusion. The dotted black curves show the effect of donor exclusion starting at the beginning of alimentary risk. Most infected donors have no transfusion history and cannot, therefore, be excluded from blood donation.

Finally, we considered the absolute prevalence of infected donors according to their history of blood transfusion (Figure 5). Most infected donors do not have a transfusion history, which explains the negligible effect of a policy excluding transfusion recipients from donation.

To determine whether the same model could also predict transition into a positive endemic equilibrium of the infection, we made the unrealistic assumptions that the rates of donor recruitment and donor loss are constant between the ages of 18 and 67 and that the rate of receiving a blood transfusion is constant throughout life. Then the model showed an extremely long time (>2,000 years) before positive equilibrium would be reached (results not shown).

## Discussion

Our model is the first attempt to describe in a realistic way the transmission of infections through blood transfusions. In 1994, Velasco-Hernández proposed a model for the spread of Chagas disease by vectors and blood transfusion (*13*). His model was used by Roberts and Heesterbeek to introduce their new concept to estimate the effort to eradicate an infectious disease (*14*). Huang and Villasana included transmission through blood transfusion in an AIDS model (*15*). All these models have in common what Inaba and Sekine state about their extension of Velasco-Hernández’s Chagas model: “…here we assume that blood donors are randomly chosen from the total population, and so there is no screening and the recipients of blood donations are donating blood themselves at the same rate as anybody else. This is an unrealistic assumption, but we will use it.” (*16*). These models implicitly describe transmission through blood transfusion exactly like person-to-person transmission by droplet infections.

The key innovation in our model is the simultaneous incorporation of 6 functions that all depend explicitly on the age of a person: 1) natural death rate, 2) rate of receiving a blood transfusion, 3) rates of donor recruitment, 4) donor loss, 5) death rate associated with transfusions, and 6) proportion of transfusion recipients at increased risk for death. The age-dependent effects of these processes cannot be ignored. Peak ages of donor activity (≈22 years) and of receiving a blood transfusion (≈70 years) are quite distinct and ≈50 years apart. This age pattern does not favor the spread of infection by blood transfusion. Another factor that acts against the infection becoming endemic is the transfusion-associated death rate. The good quality of the follow-up data of nearly 3,000 patients helped to incorporate realistic assumptions about the survival probabilities of transfusion recipients (*4*). The only data available about the joint distribution of blood donor activity and history of a blood transfusion was the CJD case-control study performed in Göttingen, Germany (*7*).

The length of the incubation period plays a major role in transmission dynamics and hence was subject to a sensitivity analysis. The model does not account for possible changes of infectivity during the incubation period. The model represents a worst-case scenario because it assumes 100% infectivity throughout the period of infection. Even under this extreme assumption, donor exclusion can prevent only 0.9% (or 5%) of the expected deaths, assuming the incubation period has a mean duration of 16 (or 50) years. The main explanation for this surprising result is that most infected donors have been infected by the alimentary route and never received any blood transfusion and, therefore, are not eligible for donor exclusion.

The present simulations have arbitrarily assumed a cumulative incidence of alimentary infection, about 25 per million (2,000 per 80 million). With pessimistic assumptions, the model predicts either 19.5 deaths per million for the short incubation period or 9 deaths per million for the long incubation period in the absence of spread through blood transfusion. This corresponds to at least 9 (36%) of 25 deaths attributable to the infection, which is ≈2 orders of magnitude higher than expected for vCJD in the United Kingdom. As of July 2006, the number of vCJD cases in the United Kingdom was 160. If we assume that the total number of cases will be 200, then our assumption corresponds to about 3.3 cases per million. Thus, at most, 1.4% of infected persons would die from the infection (unless a second wave of vCJD cases with a long incubation period occurs). According to our model, 0.9% of the deaths could be prevented by donor exclusion under the assumption of the short incubation period. In absolute numbers this would be ≈2 cases.

In France, the total number of vCJD cases recorded through July 2006 is 18. Even under the assumption that this number represents only 35% of the total number of cases (*17*), the absolute expected number of prevented cases would be <1. In 1998, France decided to exclude donors with a transfusion history, primarily to reduce the spread of viruses. The present model could be modified to assess the effectiveness of excluding donors with transfusion history for preventing emerging infections with different modes of transmission and additional epidemiologic states, e.g., latent or immune.

Our worst-case scenario assumptions of the epidemiology might seem similar to the situation in the United Kingdom. In Germany, no case of vCJD has been reported, which indicates that the expected number of cases in Germany is at least 2 orders of magnitude less than that in the United Kingdom. This latter aspect was considered in the interpretation of our model by a working group commissioned by the German Federal Minister of Health, which recommended in April 2006 that persons with a transfusion history not be excluded from donating blood (*18*). Our analysis enables different countries to perform their own risk assessment and choose a strategy according to the absolute number of cases observed or expected.

## Supplementary Material

Technical Appendix Blood Transfusion and Spread of Variant Creutzfeldt-Jakob Disease
