# Supplementary material for: Blood Transfusion and Spread of Variant Creutzfeldt-Jakob Disease
Source: Emerg Infect Dis. 2007 Jan;13(1):89–96. doi: 10.3201/eid1301.060396 (PMC2725807; doi:10.3201/eid1301.060396)
Supplement: Technical Appendix — Blood Transfusion and Spread of Variant Creutzfeldt-Jakob Disease [file 06-0396_Techapp-s1.pdf]

This material, provided by the authors as a supplement to Blood Transfusion and Spread of Variant Creutzfeldt-Jakob Disease, is not part of Emerging Infectious Diseases contents.

# Blood Transfusion and Spread of Variant Creutzfeldt-Jakob Disease

Klaus Dietz, Günter Raddatz, Jonathan Wallis, Norbert Müller, Inga Zerr, Hans-Peter Duerr, Hans Lefèvre , Erhard Seifried, Johannes Löwer

## Technical Appendix

### *Modelling the age-specific mortality:*

These death rates are described as a sum of three parametric functions which refer to (i) infancy and early childhood, (ii) late childhood and early adulthood, and (iii) the remaining life (a function which corresponds to a Gompertz model of aging with exponentially increasing death rate) (*1*). This idea of expressing the mortality throughout the whole life analytically is well-known since more than 100 years (Figure S 1) (*2*).

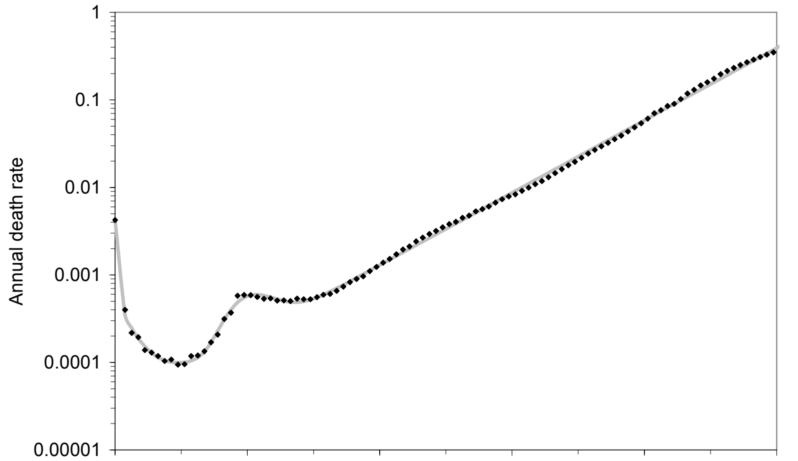


Figure S 1: Age-specific death rates in the general population. Dots: observed values; line: fitted death rates according to a parametric model involving three functions as described in the sections about demography. Beyond the age of 40 years the observations are well described by a Gompertz model which predicts that the death rates increase exponentially corresponding to a straight line on the logarithmic scale which is used here. The death rates for males and females are averaged in the present model.

The death rate initially declines very fast and reaches a minimum around the age of 10 years. Afterwards there is a unimodal peak which is well described by a lognormal distribution with a peak around 21 years. The corresponding life table, i.e. the probability of surviving a given age yields a life expectancy of 79 years (Figure S 2).

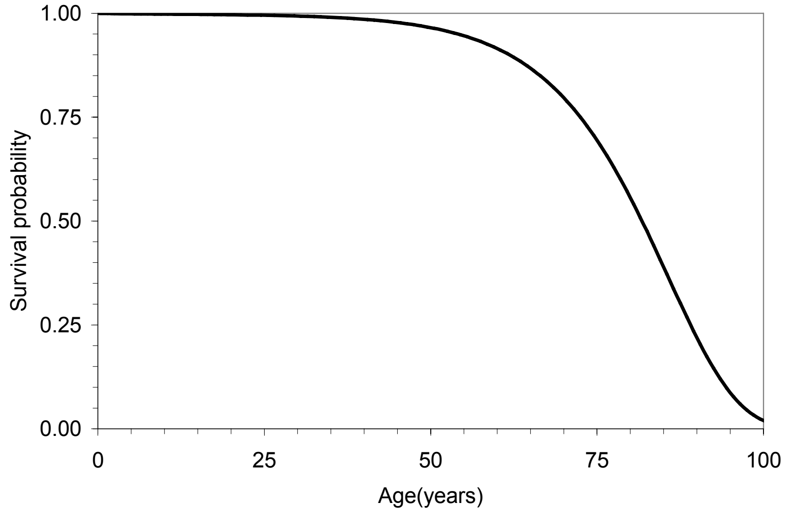


Figure S 2: Life table for the stationary population used in the present model. This corresponds to the age-distribution in the absence of the infection

The three functions $\mu_k$ describe the age-specific mortality during infancy and early childhood ($k$=0), during late childhood and early adulthood ($k$=1) and during late adulthood ($k$=2).

$$\mu_B(a) = \mu_0(a) + \mu_1(a) + \mu_2(a)$$
$$\mu_0(a) = \exp(\alpha_0)\beta_0\delta a^{\delta-1}\exp(-\beta_0 a^{\delta})$$
$$\mu_1(a) = \frac{\beta_1}{\sqrt{2\pi\sigma}}\exp\left[-\frac{1}{2}\frac{\left(\log\frac{a}{A}\right)^2}{\sigma^2}\right]$$
$$\mu_2(a) = \beta_2\exp(\beta_3 a)$$

The following parameter values were fitted by nonlinear least squares regression to the age-specific mortality of Germany, where the data from both sexes were averaged using the age specific survival function as weights (http://www.destatis.de/download/d/bevoe/sterbet04.xls).

| Parameter | Value |
|---|---|
| $\alpha_0$ | -6.315 |
| $\beta_0$ | 0.4425 |
| $\delta$ | 0.7939 |
| $\beta_1$ | 0.0001649 |
| $\sigma$ | 0.1716 |
| $A$ | 20.61 |
| $\beta_2$ | 0.00002867 |
| $\beta_3$ | 0.09524 |

Each year a fixed number of susceptible newborns is added to the population, such that the total size of the population is about 80 million.

### *Modelling donor activities*

The yearly rate of becoming an active donor (the donor recruitment rate) is denoted by $\rho(a)$. It is zero below 18 and above 67 years. The age-specific number of first time donors of the German Red Cross (DRK) Blood Service was divided by the corresponding age-specific number of individuals in the population as provided by the Federal Statistical Office (to obtain an age specific rate of becoming a new active donor. This rate has to be multiplied by an age-independent scaling factor which ensures that the calculated total number of active donors corresponds to the observed number of about 2.5 million. The logarithm of the empirical yearly rate is approximated by a polynomial of fifth degree with the following parameters:

$$\rho(a) = 10^{\left(r_0 + r_1 a + r_2 (a-42)^2 + r_3 (a-42)^3 + r_4 (a-42)^4 + r_5 (a-42)^5\right)} \quad \text{for} \quad 18 \le a \le 67$$

| Parameter | Value |
|---|---|
| $r_0$ | -2.96 |
| $r_1$ | -0.0145 |
| $r_2$ | -0.0015 |
| $r_3$ | 0.0000784 |
| $r_4$ | 0.0000023 |
| $r_5$ | -0.000000236 |

The rate of terminating active donation is denoted by $\sigma(a)$. Depending on the age of recruitment we estimate the average time as active donor by fitting an exponential distribution to the age-distribution of active donors. For these age specific average

sojourn times we fit a polynomial of fourth degree. The inverse of these averages we take as the age-specific rate $\sigma(a)$:

$$\sigma(a)=\frac{1}{s_0+s_1a+s_2(a-42.5)^2+s_3(a-42.5)^3+s_4(a-42.5)^4}$$

with parameters

| Parameter | Value |
|---|---|
| $s_0$ | 25.92 |
| $s_1$ | -0.38 |
| $s_2$ | -0.00254 |
| $s_3$ | 0.000289 |
| $s_4$ | -0.000006 |

The donor recruitment rate is estimated from the age distribution of first-time donors in our sample (Figure S 3A) and from the age distribution of the population (Figure S 3B). The age-specific ratios of these frequencies are multiplied by a scaling factor in order to obtain a realistic size of the donor population. The recruitment rate is highest at the age of 18 years, declines by more than one order of magnitude up to the age of 28, remains almost constant up to the age of 50 and declines substantially in aged donors (Figure S 3C).

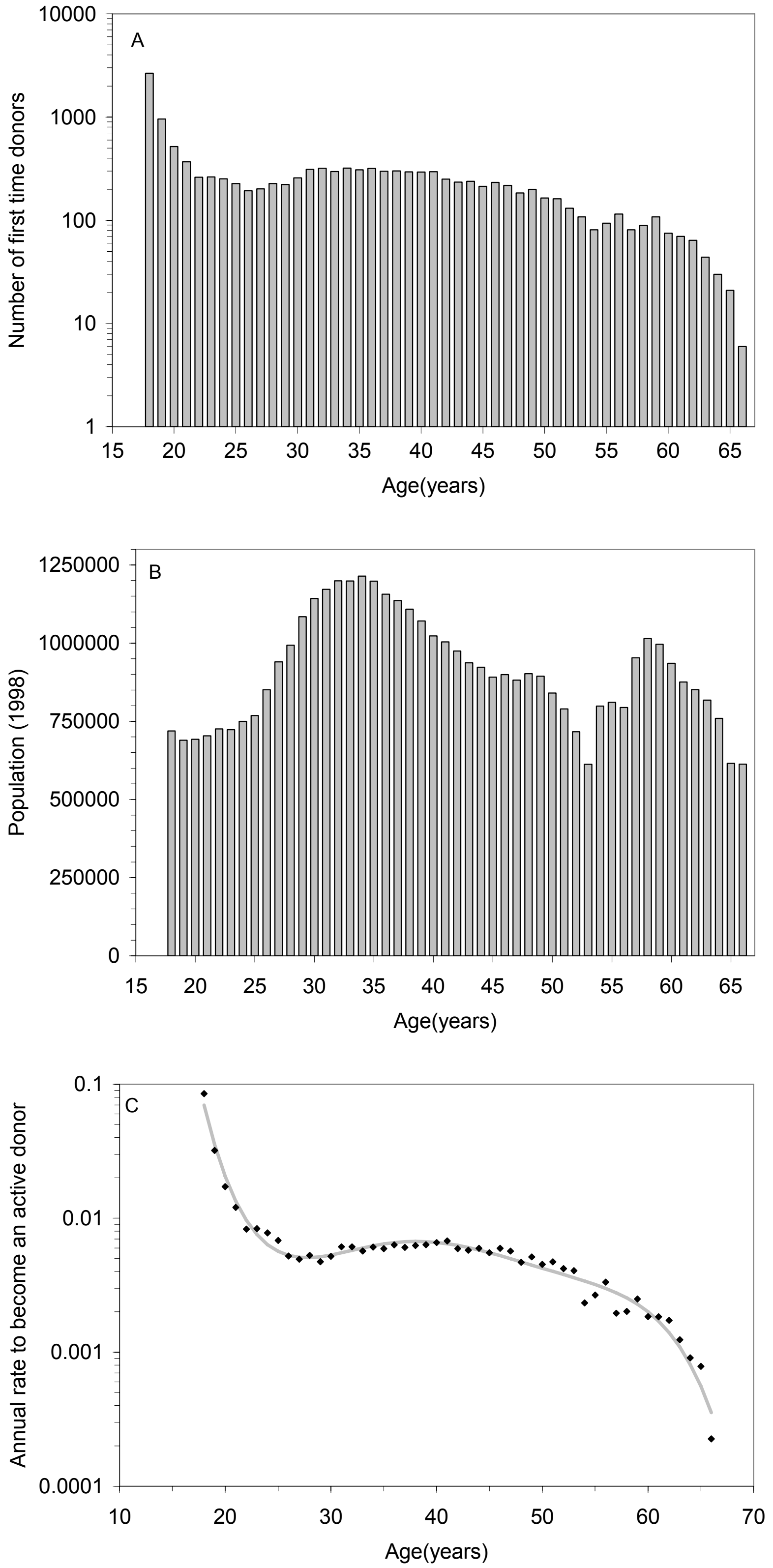


Figure S 3: Estimation of the rate of becoming an active donor. A) Age-distribution of first-time donors, B) Age-distribution of the population of Germany C) observed (dots) and fitted (line) logarithm of the age-specific rate to become an active donor.

Donor loss is estimated from the age distributions of active donors as a function of the youngest age as active donor. An exponential distribution is fitted to each of these distributions for all yearly age classes from 18 to 66 years (Figure S 4A provides an example). The corresponding mean durations are fitted by a polynomial (Figure S 4B).

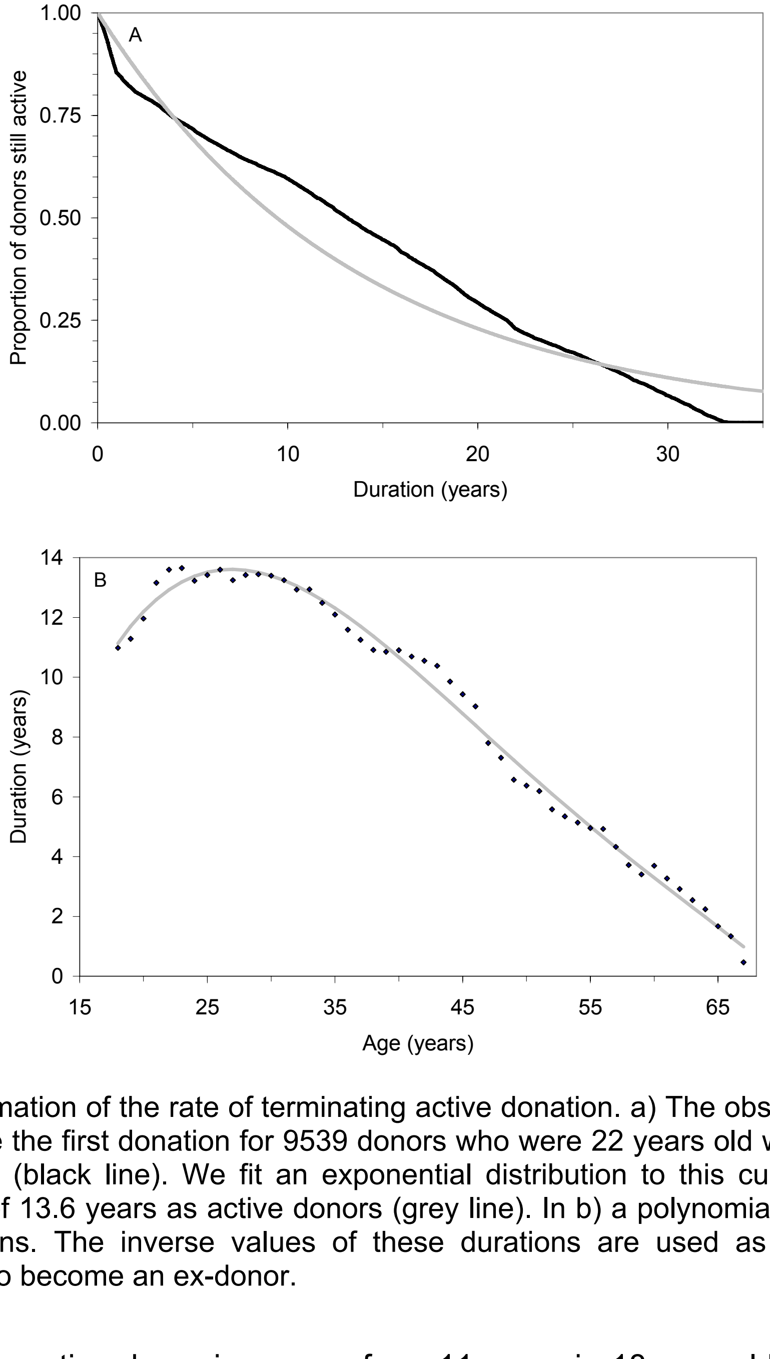


Figure S 4: Estimation of the rate of terminating active donation. a) The observed distribution of the time since the first donation for 9539 donors who were 22 years old when they started to donate blood (black line). We fit an exponential distribution to this curve and obtain a mean duration of 13.6 years as active donors (grey line). In b) a polynomial is fitted to these average durations. The inverse values of these durations are used as the age-specific transition rates to become an ex-donor.

The duration as active donor increases from 11 years in 18 year old donors to 13.7 years in 23 year old donors, and subsequently declines nearly linearly to zero until the maximum age of active donors.

### *Modelling the age-specific prevalence of active donors*

The estimates of donor recruitment and loss of donors produces the age-specific prevalence of active donors (Figure S 5). The prevalence peaks at ≈24 years of age and subsequently declines monotonically to zero by age 67. The overall prevalence in the population is 3%, i.e., 2.4 million donors in a population of ≈80 million.

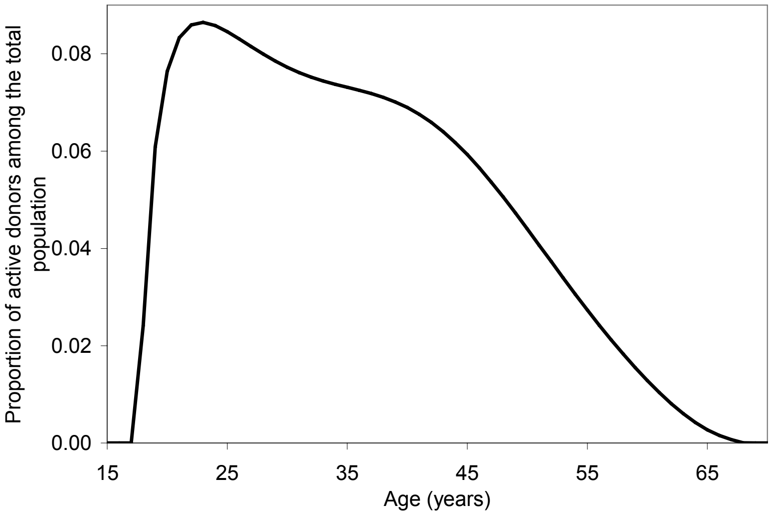


Figure S 5: Predicted age-specific prevalence of active blood donors, calculated on the basis of the parameter estimates of the rates of donor recruitment and donor loss.

### *Modelling the risk of receiving transfusions*

The yearly age-specific risk of receiving a transfusion $\varepsilon_B(a)$ among the fraction $b_\varepsilon$ of the population with a positive transfusion risk is estimated from the age-specific transfusion risk at the University Hospital Essen, Germany during one month. The function has a peak at birth which declines exponentially and another peak at about 65 years of age:

$$\varepsilon_B(a) = \mu_0 + \mu_1 \exp\left(-\mu_2 a\right) + \frac{\mu_3}{\sigma} \exp\left[-\frac{\left(a-\mu_4\right)^2}{2\sigma^2}\right].$$

A graphical representation is given by Figure S 6.

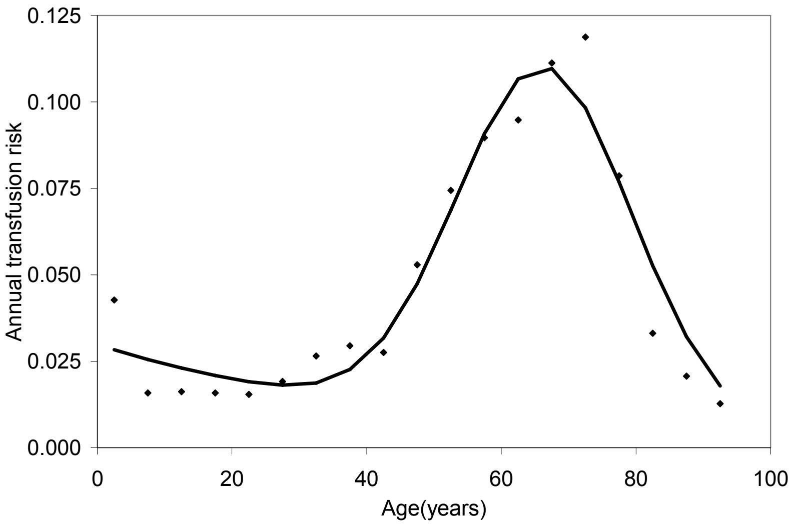


Figure S 6: The observed (dots) and fitted (line) age-specific transfusion risk.

The parameter values for the fitted function are given in the following Table:

| Parameter | Value |
|---|---|
| $\mu_0$ | 0.0155 |
| $\mu_1$ | 0.0412 |
| $\mu_2$ | 0.259 |
| $\mu_3$ | 1.133 |
| $\sigma$ | 11.16 |
| $\mu_4$ | 65.35 |

The cumulative transfusion risk is given by:

$$\mathrm{E}_B(a) = \int_0^a \varepsilon_B(s)ds \,.$$

The following expression describes the age-specific risk of transfusion for the total population:

$$\varepsilon(a) = \frac{b_\varepsilon \varepsilon_B(a)\exp(-\mathrm{E}_B(a))}{1 - b_\varepsilon(1 - \exp(-\mathrm{E}_B(a)))},$$

which is obtained by calculating the hazard rate for the proportion of individuals without transfusion in the denominator of the previous expression.

### *Modelling the transfusion associated mortality*

The proportion of transfusion recipients with increased mortality is described by the generalised logistic function: $q(a) = p_0 + \frac{p_1 - p_0}{1 + \left(\frac{A_q}{a}\right)^c}$.

Sex-specific fits are presented in Figure S 7.

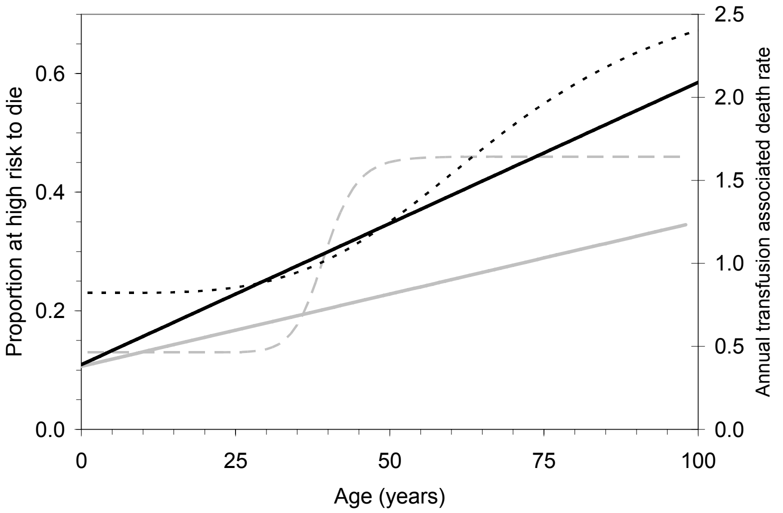


Figure S 7: Age-and sex-specific parameter values for the proportion $q(a)$ of recipients with an increased risk of death (males: dotted in black, females: dashed in grey) and the linearly increasing death rates $\beta(a)$ for those at increased risk of dying (males: solid-black, females: solid-grey).

Parameters for both sexes combined are fitted from original data (published in (*3*)), and are given in the following Table:

| Parameter | Value |
|---|---|
| $p_0$ | 0.170 |
| $p_1$ | 0.4824 |
| $A_q$ | 42.1 |
| c | 11.8 |

The generalised logistic function predicts that the proportion of transfusion recipients with increased mortality increases from 17% at birth to about 48% in old age. The point of inflection is at the age of about 42 years. The high value of nearly 12 for the exponent $c$ indicates a steep slope at the point of inflection. For those individuals with a high transfusion associated death rate this rate increases linearly with age: $\beta(a) = \beta_4 + m_\beta a$ with the following parameters

| Parameter | Value |
|---|---|
| $\beta_4$ | 0.374 |
| $m_\beta$ | 0.0125 |

This means that at birth the life expectancy for those affected by an increased death rate is about 2.5 years. This value decreases to about 0.5 years for age 80 years.

The cumulative death rate is given by $\mathrm{B}(a) = \beta_4 a + m_\beta \frac{a^2}{2}$.

The survival probability for a 3 year old girl is much closer to the survival of the general population compared to a 70 year old man (Figure S 8).

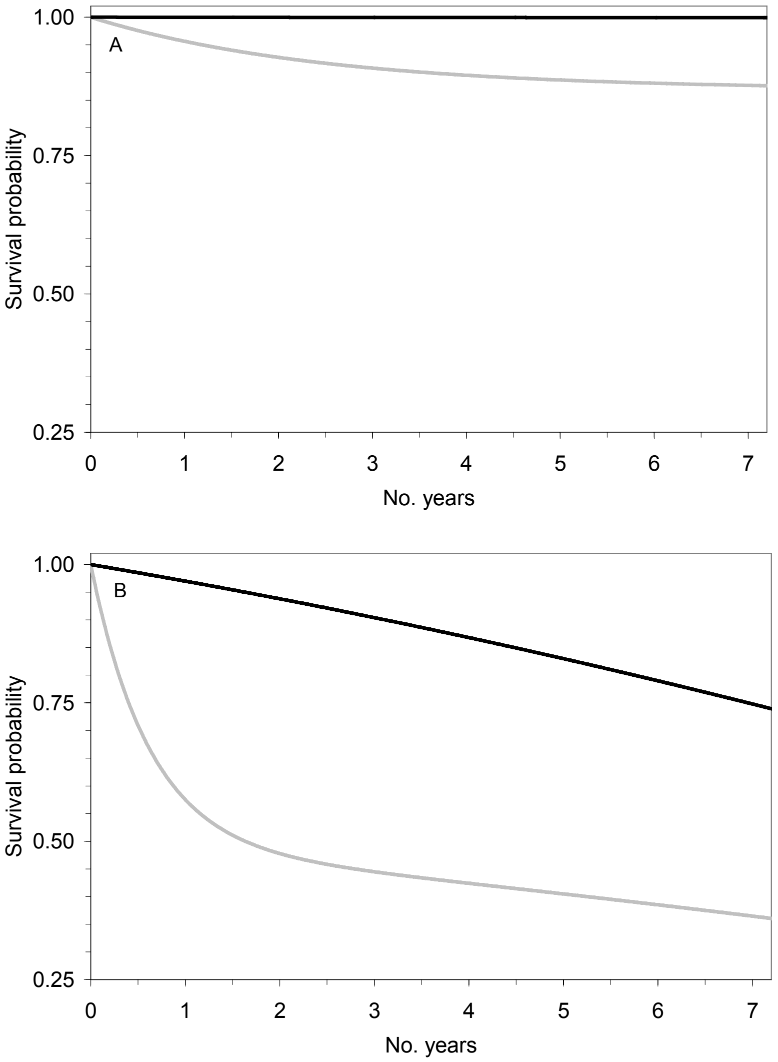


Figure S 8: The predicted survival curves (grey) for two patients together with the expected survival without transfusion-associated risk (black). A) 3 year old girl, B) 70 year old man.

Furthermore, the asymptote is approached more slowly for a 3 year old girl indicating a longer survival. The goodness of fit of the model has been evaluated by dividing the 1284 males and the 1605 females into deciles according to age such that there are about 130 and 160 individuals, respectively, in each age group (Figure S 9).

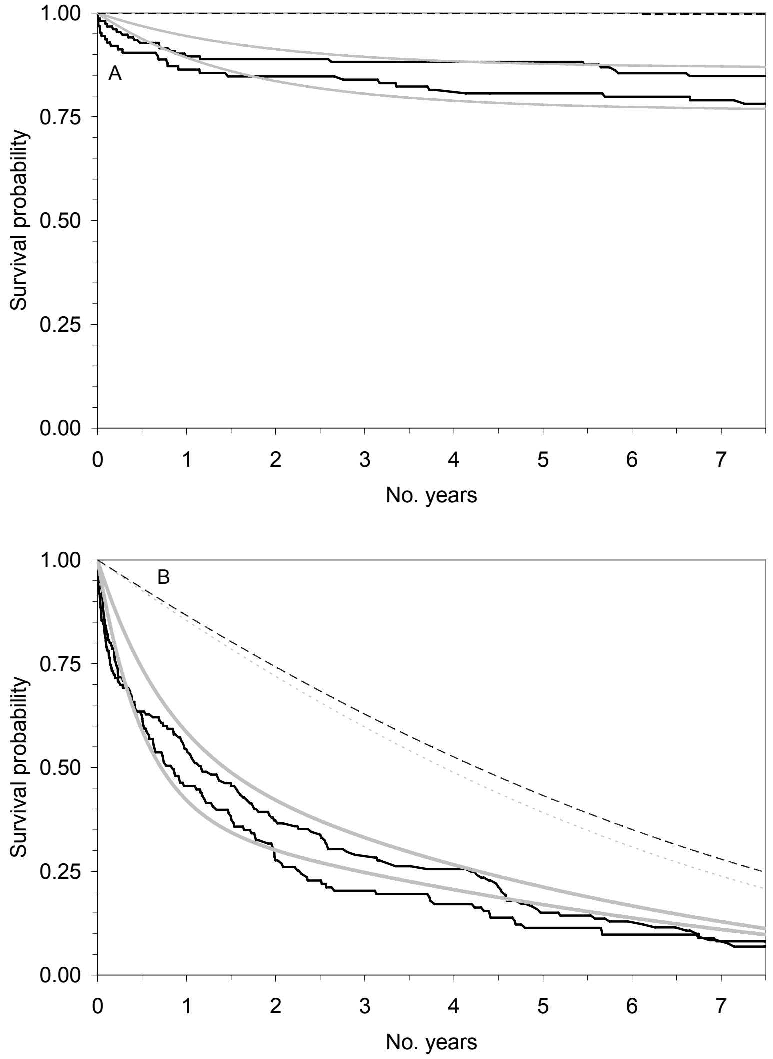


Figure S 9: Goodness of model fit for the lowest (A) and the highest (B) sex-specific age deciles of survival after a transfusion together with the expected survival of those without transfusion. The lower curves are for male, the intermediate curves for females. Black: observed, grey: predicted. The upper dashed curves show the expected survival without transfusion (males: dashed in black, females: dotted in grey; in (A) both approximate the value of one and are almost identical).

The goodness of fit of this model is further illustrated in Figure S 10:

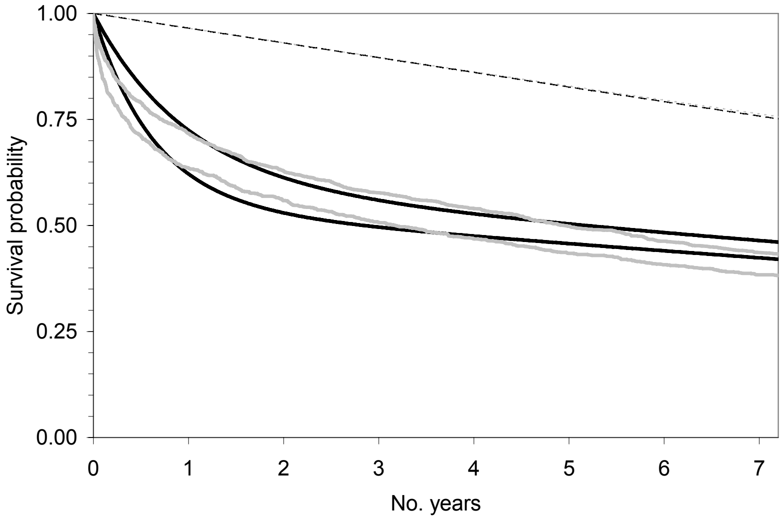


Figure S 10: Observed (grey) and predicted (black) sex-specific survival curves after a blood transfusion (all ages combined). The lower curves are for males; the intermediate curves are for females; the upper curves show the expected survival rates without transfusion (dashed for males, dotted for females; both are almost identical).

Since we model explicitly the transfusion associated death rates we have to decrease the general death rates according to the following expression in order to keep the total survival equal to the observations

$$\mu(a) = \mu_B(a) - \frac{b_\varepsilon \beta(a) \int_0^a q(s)\varepsilon_B(s) \exp\left(-\mathrm{E}_B(s) - \left(\mathrm{B}(a) - \mathrm{B}(s)\right)\right) ds}{1 - b_\varepsilon \int_0^a q(s)\varepsilon_B(s) \exp\left(-\mathrm{E}_B(s)\right)\left(1 - \exp\left(-\left(\mathrm{B}(a) - \mathrm{B}(s)\right)\right)\right) ds}.$$

### *Modelling the infection*

The sojourn time in the infected stage before an individual dies due to the infection has a gamma distribution with shape parameter $n = 16$ and $\alpha$ = 0.0625 per year which corresponds to an average duration of 16 years. This value is reduced to 0.02 to explore the sensitivity of the results with respect to this parameter. The density of the incubation time distribution is not very different from a lognormal distribution with the same mean and standard deviation (Figure S 11).

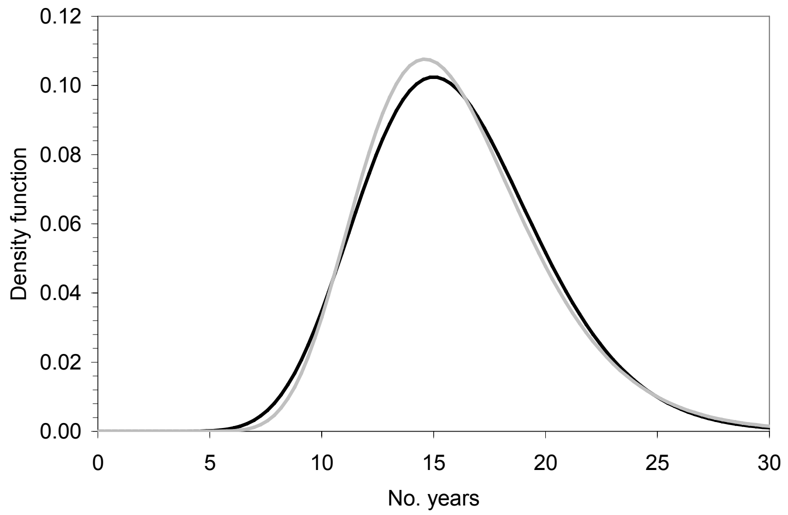


Figure S 11: Density of the gamma distributed incubation time (grey line) in comparison to a lognormal density with the same mean and variance (black line).

The risk of an infection via the alimentary route is given by the following parameters:

$\lambda(t) = \lambda_0 \quad \text{für } 0 \le t < 10,$

$\lambda(t) = 0 \quad \text{für } t \ge 10.$

The probability that a transfusion from an infected donor is infective is denoted by the parameter $f$ which is assigned to the values of zero and one.

### *Modelling donor exclusion*

Donor exclusion is modelled by two parameters: coverage $b$ and a time-dependent exclusion rate $\gamma(t)$ which allows determining the “velocity” of donor exclusion. By means of the coverage parameter the model may take into account that a donor exclusion program may not succeed to exclude 100% of potential and active donors with transfusion history. The time-dependent parameter $\gamma(t)$ allows determining the timing of a donor exclusion program in relation to the period during which there is an alimentary infection risk.

***Model Equations***

All variables $S(t,a)$ and $I(t,a)$ depend both on time $t$ and year $a$, both of which are updated every two months, i.e. we always follow a cohort by increasing simultaneously time and age by two months. All 26 variables are distinguished by appropriate indices of the numbers of susceptible ($S(t,a)$) and infective ($I(t,a)$) individuals.

Explanation of the indices of the 26 variables

- 1st index: without history of transfusion = 0 (non-recipients); with history transfusion = 1 (recipients);
- 2nd index: potential donor = 0; active donor = 1, past donor or excluded from donation = 2;
- 3rd index (for recipients, i.e. 1st index=1, and 2nd index <2): not deferrable=0, deferrable=1;
- 3rd index (for recipients, i.e. 1st index=1, and 2nd index =2): without transfusion associated risk of death = 0; with transfusion associated risk of death =1;
- 3rd index for individuals infected via the alimentary route (1st index = 0): number of the fictitious incubation state (j=1, ... , n);
- 4th index (for recipients, i.e. 1st index = 1, and 2nd index <2): without transfusion associated risk of death =0; with transfusion associated risk of death =1;
- 4th index (for recipients, i.e. 1st index = 1, and 2nd index =2) for infected individuals: number of the fictitious incubation state (j=1, ... , n);
- 5th index (for recipients, i.e. 1st index = 1, and 2nd index <2) for infected individuals: number of the fictitious incubation state (j=1, ... , n)

With this notation, the 26 variables represent proportions in the population with following attributes:

| | | 1st index | 2nd index | 3rd index | 4th index |
|---|---|---|---|---|---|
| 1 | $S_{00}$ | non-recipient | no donor | | |
| 2 | $S_{01}$ | non-recipient | donor | | |
| 3 | $S_{02}$ | non-recipient | ex-donor | | |
| 4 | $S_{1000}$ | recipient | no donor | deferrable | no extra mortality |
| 5 | $S_{1001}$ | recipient | no donor | deferrable | extra mortality |
| 6 | $S_{1010}$ | recipient | no donor | not deferrable | no extra mortality |
| 7 | $S_{1011}$ | recipient | no donor | not deferrable | extra mortality |
| 8 | $S_{1100}$ | recipient | donor | deferrable | no extra mortality |
| 9 | $S_{1101}$ | recipient | donor | deferrable | extra mortality |
| 10 | $S_{1110}$ | recipient | donor | not deferrable | no extra mortality |
| 11 | $S_{1111}$ | recipient | donor | not deferrable | extra mortality |
| 12 | $S_{120}$ | recipient | ex-donor | no extra mortality | |
| 13 | $S_{121}$ | recipient | ex-donor | extra mortality | |
| 14 | $I_{00j}$ | non-recipient | no donor | | |
| 15 | $I_{01j}$ | non-recipient | donor | | |
| 16 | $I_{02j}$ | non-recipient | ex-donor | | |
| 17 | $I_{1000j}$ | recipient | no donor | not deferrable | no extra mortality |
| 18 | $I_{1001j}$ | recipient | no donor | not deferrable | extra mortality |
| 19 | $I_{1010j}$ | recipient | no donor | deferrable | no extra mortality |
| 20 | $I_{1011j}$ | recipient | no donor | deferrable | extra mortality |
| 21 | $I_{1100j}$ | recipient | donor | not deferrable | no extra mortality |
| 22 | $I_{1101j}$ | recipient | donor | not deferrable | extra mortality |
| 23 | $I_{1110j}$ | recipient | donor | deferrable | no extra mortality |
| 24 | $I_{1111j}$ | recipient | donor | deferrable | extra mortality |
| 25 | $I_{120(j-1)}$ | recipient | ex-donor | no extra mortality | |
| 26 | $I_{121j}$ | recipient | ex-donor | extra mortality | |

For the indices we use the so called "+"-Notation: if an index is replaced by a "+", then this means that we have summed all variables with different indices at this position.

The model is illustrated with Figure S 12:

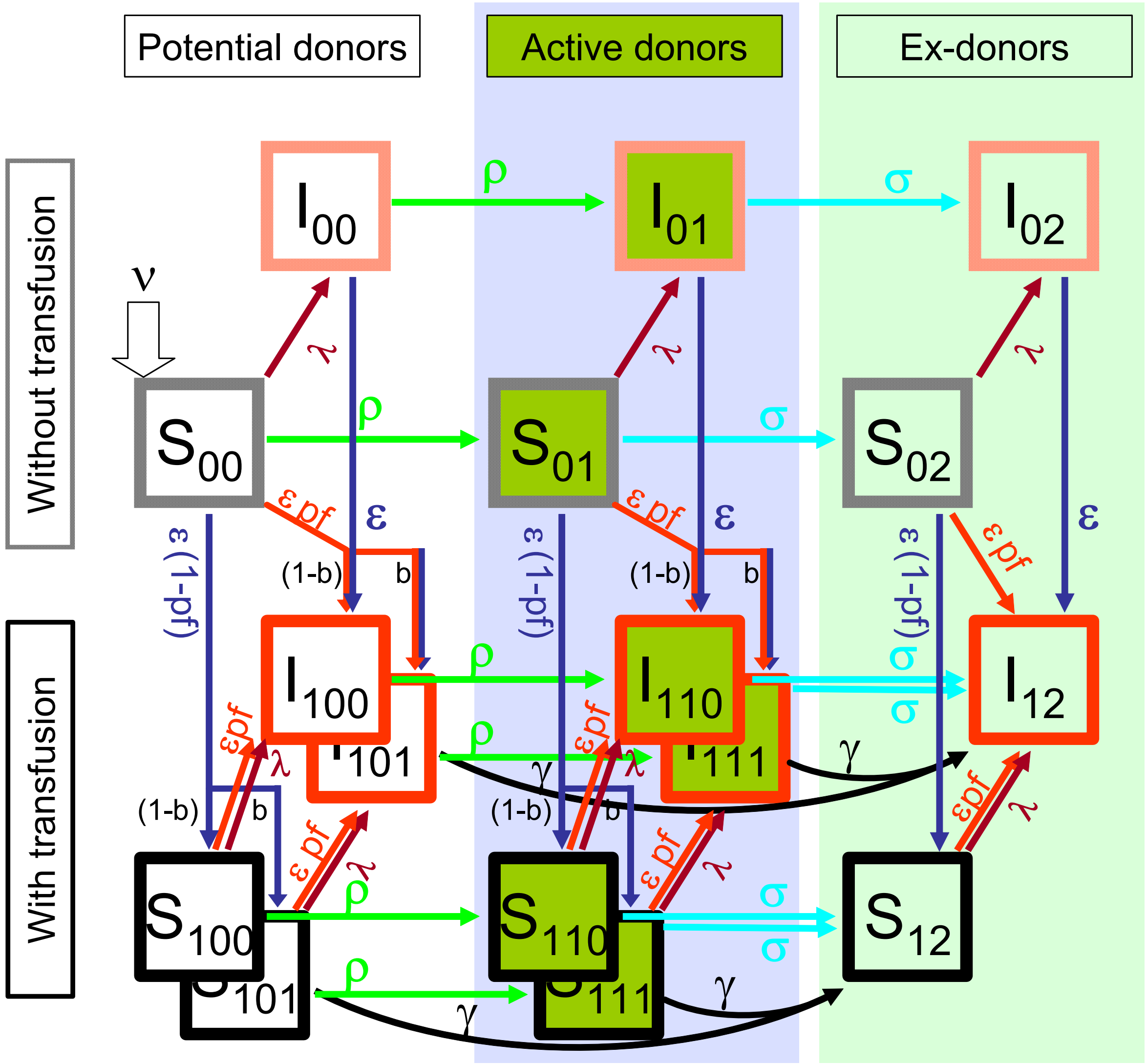


Figure S 12: States and transitions of the epidemiological model. For the transfusion recipients (all states for which the 1st index equals 1) the model distinguishes individuals with and without increased risk of dying. In the model equations this aspect is expressed with the last index of the variables. In this figure this aspect is not included. The death rates associated with each state are also not shown. Parameters: $\nu$: birth rate, $\lambda$: rate of infection during the period of alimentary transmission, $\varepsilon$ : rate of receiving blood transfusions, $\rho$: donor recruitment rate, $\sigma$: donor loss rate, $\gamma$: rate of donor exclusion, *b*: proportion of deferrable donors with transfusion history, *p*: proportion of infected donors among all donors, *f*: probability that an infected blood transfusion leads to infection of the recipient.

## Model equations

Since our step size is two months and some rates are high we use the exponential function in all equations for calculating how many individuals are still in the initial state after two months. The argument of the exponential function is the sum of all rates which describe a transition out of the corresponding variable. This approach avoids negative variables which would arise when more individuals are removed than were present at the beginning of the interval which could happen for large rates. E.g. in the second equation (for the active susceptible donors without transfusion history) we use the theory of competing risks to determine which proportion of the potential donors are added to this category: in the denominator we add all transition rates and in the numerator we select the relevant transition rate out of those from the numerator. The number of individuals in the previous state is multiplied by the probability to survive the previous year and the probability to make a transition which is the complement to the probability to make no transition.

The following expression is the time-dependent prevalence of infected active donors among all active donors:

$$p(t)=\frac{\sum_{a=18}^{67}\left[I_{01+}(a,t)+I_{11+++}(a,t)\right]}{\sum_{a=18}^{67}\left[I_{01+}(a,t)+I_{11+++}(a,t)+S_{01}(a,t)+S_{11++}(a,t)\right]}.$$

If the duration of an infection is short, i.e. the parameter $\alpha$ is high, then it may happen that a newly infected individual at the end of the first iteration step is not only in the first fictitious state of the infection but already in the second state or even in a higher state or with a small probability the individual may even have died during the iteration step. The following expressions determine the probabilities according to a gamma-distribution of finding a newly infected individual in state $j=1,...,n.$ The state $j=n+1$ corresponds to the death of the individual due to the infection:

$$q_j=\exp(-n\alpha)\frac{(n\alpha)^{j-1}}{(j-1)!},\ j=1,....,n,$$

$$q_{n+1}=1-\sum_{j=1}^{n}q_j.$$

(1) $S_{00}(t+1,a+1)=S_{00}(t,a)\exp\{-[\lambda(t)+\rho(a)+\varepsilon(a)+\mu(a)]\}$

(2) $S_{01}(t+1,a+1)=S_{01}(t,a)\exp\{-[\lambda(t)+\sigma(a)+\varepsilon(a)+\mu(a)]\}+$
$\frac{\rho(a)}{\lambda(t)+\rho(a)+\varepsilon(a)}S_{00}(t,a)\exp(-\mu(a))\langle 1-\exp\{-[\lambda(t)+\rho(a)+\varepsilon(a)]\}\rangle$

(3) $S_{02}(t+1,a+1)=S_{02}(t,a)\exp\{-[\lambda(t)+\varepsilon(a)+\mu(a)]\}+$
$\frac{\sigma(a)}{\lambda(t)+\sigma(a)+\varepsilon(a)}S_{01}(t,a)\exp(-\mu(a))\langle 1-\exp\{-[\lambda(t)+\sigma(a)+\varepsilon(a)]\}\rangle$

(4) $S_{1000}(t+1,a+1)=S_{1000}(t,a)\exp\{-[\lambda(t)+\rho(a)+fp(t)\varepsilon(a)+\mu(a)]\}+$
$\frac{\varepsilon(a)(1-fp(t))(1-b)(1-q(a))}{\lambda(t)+\rho(a)+\varepsilon(a)}S_{00}(t,a)\exp(-\mu(a))\langle 1-\exp\{-[\lambda(t)+\rho(a)+\varepsilon(a)]\}\rangle$

(5) $S_{1001}(t+1,a+1)=S_{1001}(t,a)\exp\{-[\lambda(t)+\rho(a)+fp(t)\varepsilon(a)+\mu(a)+\beta(a)]\}+$
$\frac{\varepsilon(a)(1-fp(t))(1-b)q(a)}{\lambda(t)+\rho(a)+\varepsilon(a)}S_{00}(t,a)\exp(-\mu(a))\langle 1-\exp\{-[\lambda(t)+\rho(a)+\varepsilon(a)]\}\rangle$

(6) $S_{1010}(t+1,a+1)=S_{1010}(t,a)\exp\{-[\lambda(t)+\rho(a)+fp(t)\varepsilon(a)+\gamma(t)+\mu(a)]\}+$
$\frac{\varepsilon(a)(1-fp(t))b(1-q(a))}{\lambda(t)+\rho(a)+\varepsilon(a)}S_{00}(t,a)\exp(-\mu(a))\langle 1-\exp\{-[\lambda(t)+\rho(a)+\varepsilon(a)]\}\rangle$

(7) $S_{1011}(t+1,a+1)=S_{1011}(t,a)\exp\{-[\lambda(t)+\rho(a)+fp(t)\varepsilon(a)+\gamma(t)+\mu(a)+\beta(a)]\}+$
$\frac{\varepsilon(a)(1-fp(t))bq(a)}{\lambda(t)+\rho(a)+\varepsilon(a)}S_{00}(t,a)\exp(-\mu(a))\langle 1-\exp\{-[\lambda(t)+\rho(a)+\varepsilon(a)]\}\rangle$

(8) $S_{1100}(t+1,a+1)=S_{1100}(t,a)\exp\{-[\lambda(t)+\sigma(a)+fp(t)\varepsilon(a)+\mu(a)]\}+$
$\frac{\rho(a)}{\lambda(t)+\rho(a)+fp(t)\varepsilon(a)}S_{1000}(t,a)\exp(-\mu(a))\langle 1-\exp\{-[\lambda(t)+\rho(a)+fp(t)\varepsilon(a)]\}\rangle+$
$\frac{\varepsilon(a)(1-fp(t))(1-b)(1-q(a))}{\lambda(t)+\sigma(a)+\varepsilon(a)}S_{01}(t,a)\exp(-\mu(a))\langle 1-\exp\{-[\lambda(t)+\sigma(a)+\varepsilon(a)]\}\rangle$

(9) $S_{1101}(t+1,a+1)=S_{1101}(t,a)\exp\{-[\lambda(t)+\sigma(a)+fp(t)\varepsilon(a)+\beta(a)+\mu(a)]\}+$
$\frac{\rho(a)}{\lambda(t)+\rho(a)+fp(t)\varepsilon(a)}S_{1001}(t,a)\exp(-\mu(a)-\beta(a))\langle 1-\exp\{-[\lambda(t)+\rho(a)+fp(t)\varepsilon(a)]\}\rangle+$
$\frac{\varepsilon(a)(1-fp(t))(1-b)q(a)}{\lambda(t)+\sigma(a)+\varepsilon(a)}S_{01}(t,a)\exp(-\mu(a))\langle 1-\exp\{-[\lambda(t)+\sigma(a)+\varepsilon(a)]\}\rangle$

(10) $S_{1110}(t+1,a+1)=S_{1110}(t,a)\exp\{-[\lambda(t)+\sigma(a)+fp(t)\varepsilon(a)+\gamma(t)+\mu(a)]\}+$

$\frac{\rho(a)}{\lambda(t)+\rho(a)+fp(t)\varepsilon(a)+\gamma(t)}S_{1010}(t,a)\exp(-\mu(a))\langle 1-\exp\{-[\lambda(t)+\rho(a)+fp(t)\varepsilon(a)+\gamma(t)]\}\rangle+$

$\frac{\varepsilon(a)(1-fp(t))b(1-q(a))}{\lambda(t)+\sigma(a)+\varepsilon(a)}S_{01}(t,a)\exp(-\mu(a))\langle 1-\exp\{-[\lambda(t)+\sigma(a)+\varepsilon(a)]\}\rangle$

(11) $S_{1111}(t+1,a+1)=S_{1111}(t,a)\exp\{-[\lambda(t)+\sigma(a)+fp(t)\varepsilon(a)+\gamma(t)+\mu(a)+\beta(a)]\}+$

$\frac{\rho(a)}{\lambda(t)+\rho(a)+fp(t)\varepsilon(a)+\gamma(t)}S_{1011}(t,a)\exp(-\mu(a)-\beta(a))\langle 1-\exp\{-[\lambda(t)+\rho(a)+fp(t)\varepsilon(a)+\gamma(t)]\}\rangle+$

$\frac{\varepsilon(a)(1-fp(t))bq(a)}{\lambda(t)+\sigma(a)+\varepsilon(a)}S_{01}(t,a)\exp(-\mu(a))\langle 1-\exp\{-[\lambda(t)+\sigma(a)+\varepsilon(a)]\}\rangle$

(12) $S_{120}(t+1,a+1)=S_{120}(t,a)\exp\{-[\lambda(t)+fp(t)\varepsilon(a)+\mu(a)]\}+$

$\frac{\varepsilon(a)(1-fp(t))(1-q(a))}{\lambda(t)+\varepsilon(a)}S_{02}(t,a)\exp(-\mu(a))\langle 1-\exp\{-[\lambda(t)+\varepsilon(a)]\}\rangle$

$\frac{\sigma(a)}{\lambda(t)+\sigma(a)+fp(t)\varepsilon(a)}S_{1100}(t,a)\exp(-\mu(a))\langle 1-\exp\{-[\lambda(t)+\sigma(a)+fp(t)\varepsilon(a)]\}\rangle+$

$\frac{\sigma(a)+\gamma(t)}{\lambda(t)+\sigma(a)+fp(t)\varepsilon(a)+\gamma(t)}S_{1110}(t,a)\exp(-\mu(a))\langle 1-\exp\{-[\lambda(t)+\sigma(a)+fp(t)\varepsilon(a)+\gamma(t)]\}\rangle$

$\frac{\gamma(t)}{\lambda(t)+\rho(a)+fp(t)\varepsilon(a)+\gamma(t)}S_{1010}(t,a)\exp(-\mu(a))\langle 1-\exp\{-[\lambda(t)+\rho(a)+fp(t)\varepsilon(a)+\gamma(t]\}\rangle$

(13) $S_{121}(t+1,a+1)=S_{121}(t,a)\exp\{-[\lambda(t)+fp(t)\varepsilon(a)+\mu(a)+\beta(a)]\}+$

$\frac{\varepsilon(a)(1-fp(t))q(a)}{\lambda(t)+\varepsilon(a)}S_{02}(t,a)\exp(-\mu(a))\langle 1-\exp\{-[\lambda(t)+\varepsilon(a)]\}\rangle$

$\frac{\sigma(a)}{\lambda(t)+\sigma(a)+fp(t)\varepsilon(a)}S_{1101}(t,a)\exp(-\mu(a)-\beta(a))\langle 1-\exp\{-[\lambda(t)+\sigma(a)+fp(t)\varepsilon(a)]\}\rangle+$

$\frac{\sigma(a)+\gamma(t)}{\lambda(t)+\sigma(a)+fp(t)\varepsilon(a)+\gamma(t)}S_{1111}(t,a)\exp(-\mu(a)-\beta(a))\langle 1-\exp\{-[\lambda(t)+\sigma(a)+fp(t)\varepsilon(a)+\gamma(t)]\}\rangle$

$\frac{\gamma(t)}{\lambda(t)+\rho(a)+fp(t)\varepsilon(a)+\gamma(t)}S_{1011}(t,a)\exp(-\mu(a)-\beta(a))\langle 1-\exp\{-[\lambda(t)+\rho(a)+fp(t)\varepsilon(a)+\gamma(t)]\}\rangle$

(14) $I_{00j}(t+1,a+1)=I_{00j}(t,a)\exp\left\{-\left[\rho(a)+\varepsilon(a)+\mu(a)+n\alpha\right]\right\}+$
$\frac{n\alpha}{\rho(a)+\varepsilon(a)+n\alpha}I_{00(j-1)}(t,a)\exp(-\mu(a))\left\langle 1-\exp\left\{-\left[\rho(a)+\varepsilon(a)+n\alpha\right]\right\}\right\rangle+$
$\frac{\lambda(t)}{\lambda(t)+\rho(a)+\varepsilon(a)}S_{00}(t,a)\exp(-\mu(a))\left\langle 1-\exp\left\{-\left[\lambda(t)+\rho(a)+\varepsilon(a)\right]\right\}\right\rangle q_j$

(15) $I_{01j}(t+1,a+1)=I_{01j}(t,a)\exp\left\{-\left[\sigma(a)+\varepsilon(a)+\mu(a)+n\alpha\right]\right\}+$
$\frac{n\alpha}{\sigma(a)+\varepsilon(a)+n\alpha}I_{01(j-1)}(t,a)\exp(-\mu(a))\left\langle 1-\exp\left\{-\left[\sigma(a)+\varepsilon(a)+n\alpha\right]\right\}\right\rangle+$
$\frac{\rho(a)}{\rho(a)+\varepsilon(a)+n\alpha}I_{00j}(t,a)\exp(-\mu(a))\left\langle 1-\exp\left\{-\left[\rho(a)+\varepsilon(a)+n\alpha\right]\right\}\right\rangle+$
$\frac{\lambda(t)}{\lambda(t)+\sigma(a)+\varepsilon(a)}S_{01}(t,a)\exp(-\mu(a))\left\langle 1-\exp\left\{-\left[\lambda(t)+\sigma(a)+\varepsilon(a)\right]\right\}\right\rangle q_j$

(16) $I_{02j}(t+1,a+1)=I_{02j}(t,a)\exp\left\{-\left[\varepsilon(a)+\mu(a)+n\alpha\right]\right\}+$
$\frac{\sigma(a)}{\sigma(a)+\varepsilon(a)+n\alpha}I_{01j}(t,a)\exp(-\mu(a))\left\langle 1-\exp\left\{-\left[\sigma(a)+\varepsilon(a)+n\alpha\right]\right\}\right\rangle+$
$\frac{n\alpha}{\varepsilon(a)+n\alpha}I_{02(j-1)}(t,a)\exp(-\mu(a))\left\langle 1-\exp\left\{-\left[\varepsilon(a)+n\alpha\right]\right\}\right\rangle+$
$\frac{\lambda(t)}{\lambda(t)+\varepsilon(a)}S_{02}(t,a)\exp(-\mu(a))\left\langle 1-\exp\left\{-\left[\lambda(t)+\varepsilon(a)\right]\right\}\right\rangle q_j$

(17) $I_{1000j}(t+1,a+1)=I_{1000j}(t,a)\exp\left\{-\left[\rho(a)+\mu(a)+n\alpha\right]\right\}+$
$\frac{n\alpha}{\rho(a)+n\alpha}I_{1000(j-1)}(t,a)\exp(-\mu(a))\left\langle 1-\exp\left\{-\left[\rho(a)+n\alpha\right]\right\}\right\rangle+$
$\frac{(1-b)fp(t)\varepsilon(a)(1-q(a))}{\lambda(t)+\rho(a)+\varepsilon(a)}S_{00}(t,a)\exp(-\mu(a))\left\langle 1-\exp\left\{-\left[\lambda(t)+\rho(a)+\varepsilon(a)\right]\right\}\right\rangle q_j+$
$\frac{fp(t)\varepsilon(a)+\lambda(t)}{\lambda(t)+\rho(a)+fp(t)\varepsilon(a)}S_{1000}(t,a)\exp(-\mu(a))\left\langle 1-\exp\left\{-\left[\lambda(t)+\rho(a)+fp(t)\varepsilon(a\right]\right\}\right\rangle q_j$
$+\frac{\varepsilon(a)(1-b)(1-q(a))}{\left[\rho(a)+\varepsilon(a)+n\alpha\right]}I_{00j}(t,a)\exp(-\mu(a))\left\langle 1-\exp\left\{-\left[\rho(a)+\varepsilon(a)+n\alpha\right]\right\}\right\rangle$

(18) $I_{1001j}(t+1,a+1)=I_{1001j}(t,a)\exp\left\{-\left[\rho(a)+\mu(a)+\beta(a)+n\alpha\right]\right\}+$
$\frac{n\alpha}{\rho(a)+n\alpha}I_{1001(j-1)}(t,a)\exp(-\mu(a)-\beta(a))\left\langle 1-\exp\left\{-\left[\rho(a)+n\alpha\right]\right\}\right\rangle+$
$\frac{(1-b)fp(t)\varepsilon(a)q(a)}{\lambda(t)+\rho(a)+\varepsilon(a)}S_{00}(t,a)\exp(-\mu(a))\left\langle 1-\exp\left\{-\left[\lambda(t)+\rho(a)+\varepsilon(a)\right]\right\}\right\rangle q_j+$
$\frac{fp(t)\varepsilon(a)+\lambda(t)}{\lambda(t)+\rho(a)+fp(t)\varepsilon(a)}S_{1001}(t,a)\exp(-\mu(a)-\beta(a))\left\langle 1-\exp\left\{-\left[\lambda(t)+\rho(a)+fp(t)\varepsilon(a)\right]\right\}\right\rangle q_j$
$+\frac{\varepsilon(a)(1-b)q(a)}{\left[\rho(a)+\varepsilon(a)+n\alpha\right]}I_{00j}(t,a)\exp(-\mu(a))\left\langle 1-\exp\left\{-\left[\rho(a)+\varepsilon(a)+n\alpha\right]\right\}\right\rangle$

(19) $I_{1010j}(t+1,a+1) = I_{1010j}(t,a)\exp\{-[\rho(a)+\gamma(t)+\mu(a)+n\alpha]\}+$

$\frac{n\alpha}{\rho(a)+\gamma(t)+n\alpha} I_{1010(j-1)}(t,a)\exp(-\mu(a))\langle 1-\exp\{-[\rho(a)+\gamma(t)+n\alpha]\}\rangle +$

$\frac{bfp(t)\varepsilon(a)(1-q(a))}{\lambda(t)+\rho(a)+\varepsilon(a)} S_{00}(t,a)\exp(-\mu(a))\langle 1-\exp\{-[\lambda(t)+\rho(a)+\varepsilon(a]\}\rangle q_j +$

$\frac{fp(t)\varepsilon(a)+\lambda(t)}{\lambda(t)+\rho(a)+fp(t)\varepsilon(a)+\gamma(t)} S_{1010}(t,a)\exp(-\mu(a))\langle 1-\exp\{-[\lambda(t)+\rho(a)+fp(t)\varepsilon(a)+\gamma(t)]\}\rangle q_j$

$\frac{\varepsilon(a)b(1-q(a))}{[\rho(a)+\varepsilon(a)+n\alpha]} I_{00j}(t,a)\exp(-\mu(a))\langle 1-\exp\{-[\rho(a)+\varepsilon(a)+n\alpha]\}\rangle$

(20) $I_{1011j}(t+1,a+1) = I_{1011j}(t,a)\exp\{-[\rho(a)+\gamma(t)+\mu(a)+\beta(a)+n\alpha]\}+$

$\frac{n\alpha}{\rho(a)+\gamma(t)+n\alpha} I_{1011(j-1)}(t,a)\exp(-\mu(a)-\beta(a))\langle 1-\exp\{-[\rho(a)+\gamma(t)+n\alpha]\}\rangle +$

$\frac{bfp(t)\varepsilon(a)q(a)}{\lambda(t)+\rho(a)+\varepsilon(a)} S_{00}(t,a)\exp(-\mu(a))\langle 1-\exp\{-[\lambda(t)+\rho(a)+\varepsilon(a)]\}\rangle q_j +$

$\frac{fp(t)\varepsilon(a)+\lambda(t)}{\lambda(t)+\rho(a)+fp(t)\varepsilon(a)+\gamma(t)} S_{1011}(t,a)\exp(-\mu(a)-\beta(a))\langle 1-\exp\{-[\lambda(t)+\rho(a)+fp(t)\varepsilon(a)+\gamma(t)]\}\rangle q_j$

$+\frac{\varepsilon(a)bq(a)}{[\rho(a)+\varepsilon(a)+n\alpha]} I_{00j}(t,a)\exp(-\mu(a))\langle 1-\exp\{-[\rho(a)+\varepsilon(a)+n\alpha]\}\rangle$

(21) $I_{1100j}(t+1,a+1) = I_{1100j}(t,a)\exp\{-[\sigma(a)+\mu(a)+n\alpha]\}+$

$\frac{n\alpha}{\sigma(a)+n\alpha} I_{1100(j-1)}(t,a)\exp(-\mu(a))\langle 1-\exp\{-[\sigma(a)+n\alpha]\}\rangle +$

$\frac{\rho(a)}{\rho(a)+n\alpha} I_{1000j}(t,a)\exp(-\mu(a))\langle 1-\exp\{-[\rho(a)+n\alpha]\}\rangle +$

$\frac{(1-b)fp(t)\varepsilon(a)(1-q(a))}{\lambda(t)+\sigma(a)+\varepsilon(a} S_{01}(t,a)\exp(-\mu(a))\langle 1-\exp\{-[\lambda(t)+\sigma(a)+\varepsilon(a)]\}\rangle q_j +$

$\frac{fp(t)\varepsilon(a)+\lambda(t)}{\lambda(t)+\sigma(a)+fp(t)\varepsilon(a)} S_{1100}(t,a)\exp(-\mu(a))\langle 1-\exp\{-[\lambda(t)+\sigma(a)+fp(t)\varepsilon(a)]\}\rangle q_j$

$+\frac{\varepsilon(a)(1-b)(1-q(a))}{[\sigma(a)+\varepsilon(a)+n\alpha]} I_{01j}(t,a)\exp(-\mu(a))\langle 1-\exp\{-[\sigma(a)+\varepsilon(a)+n\alpha]\}\rangle$

(22) $I_{1101j}(t+1,a+1) = I_{1101j}(t,a)\exp\{-[\sigma(a)+\mu(a)+\beta(a)+n\alpha]\}+$

$\frac{n\alpha}{\sigma(a)+n\alpha} I_{1101(j-1)}(t,a)\exp(-\mu(a)-\beta(a))\langle 1-\exp\{-[\sigma(a)+n\alpha]\}\rangle +$

$\frac{\rho(a)}{\rho(a)+n\alpha} I_{1001j}(t,a)\exp(-\mu(a)-\beta(a))\langle 1-\exp\{-[\rho(a)+n\alpha]\}\rangle +$

$\frac{(1-b)fp(t)\varepsilon(a)q(a)}{\lambda(t)+\sigma(a)+\varepsilon(a)} S_{01}(t,a)\exp(-\mu(a))\langle 1-\exp\{-[\lambda(t)+\sigma(a)+\varepsilon(a)]\}\rangle q_j +$

$\frac{fp(t)\varepsilon(a)+\lambda(t)}{\lambda(t)+\sigma(a)+fp(t)\varepsilon(a)} S_{1101}(t,a)\exp(-\mu(a)-\beta(a))\langle 1-\exp\{-[\lambda(t)+\sigma(a)+fp(t)\varepsilon(a)]\}\rangle q_j$

$+\frac{\varepsilon(a)(1-b)q(a)}{[\sigma(a)+\varepsilon(a)+n\alpha]} I_{01j}(t,a)\exp(-\mu(a))\langle 1-\exp\{-[\sigma(a)+\varepsilon(a)+n\alpha]\}\rangle$

(23) $I_{1110j}(t+1,a+1)=I_{1110j}(t,a)\exp\left\{-\left[\sigma(a)+\mu(a)+\gamma(t)+n\alpha\right]\right\}+$

$\frac{n\alpha}{\sigma(a)+\gamma(t)+n\alpha}I_{1110(j-1)}(t,a)\exp(-\mu(a))\left\langle 1-\exp\left\{-\left[\sigma(a)+\gamma(t)+n\alpha\right]\right\}\right\rangle+$

$\frac{\rho(a)}{\rho(a)+\gamma(a)+n\alpha}I_{1010j}(t,a)\exp(-\mu(a))\left\langle 1-\exp\left\{-\left[\rho(a)+\gamma(t)+n\alpha\right]\right\}\right\rangle+$

$\frac{bfp(t)\varepsilon(a)(1-q(a))}{\lambda(t)+\sigma(a)+\varepsilon(a}S_{01}(t,a)\exp(-\mu(a))\left\langle 1-\exp\left\{-\left[\lambda(t)+\sigma(a)+\varepsilon(a)\right]\right\}\right\rangle q_j+$

$\frac{fp(t)\varepsilon(a)+\lambda(t)}{\lambda(t)+\sigma(a)+fp(t)\varepsilon(a)+\gamma(t)}S_{1110}(t,a)\exp(-\mu(a))\left\langle 1-\exp\left\{-\left[\lambda(t)+\sigma(a)+fp(t)\varepsilon(a)+\gamma(t)\right]\right\}\right\rangle q_j$

$+\frac{\varepsilon(a)b(1-q(a))}{\left[\sigma(a)+\varepsilon(a)+n\alpha\right]}I_{01j}(t,a)\exp(-\mu(a))\left\langle 1-\exp\left\{-\left[\sigma(a)+\varepsilon(a)+n\alpha\right]\right\}\right\rangle$

(24) $I_{1111j}(t+1,a+1)=I_{1111j}(t,a)\exp\left\{-\left[\sigma(a)+\mu(a)+\beta(a)+\gamma(t)+n\alpha\right]\right\}+$

$\frac{n\alpha}{\sigma(a)+\gamma(t)+n\alpha}I_{1111(j-1)}(t,a)\exp(-\mu(a)-\beta(a))\left\langle 1-\exp\left\{-\left[\sigma(a)+\gamma(t)+n\alpha\right]\right\}\right\rangle+$

$\frac{\rho(a)}{\rho(a)+\gamma(a)+n\alpha}I_{1011j}(t,a)\exp(-\mu(a)-\beta(a))\left\langle 1-\exp\left\{-\left[\rho(a)+\gamma(t)+n\alpha\right]\right\}\right\rangle+$

$\frac{bfp(t)\varepsilon(a)q(a)}{\lambda(t)+\sigma(a)+\varepsilon(a}S_{01}(t,a)\exp(-\mu(a))\left\langle 1-\exp\left\{-\left[\lambda(t)+\sigma(a)+\varepsilon(a)\right]\right\}\right\rangle q_j+$

$\frac{fp(t)\varepsilon(a)+\lambda(t)}{\lambda(t)+\sigma(a)+fp(t)\varepsilon(a)+\gamma(t)}S_{1111}(t,a)\exp(-\mu(a)-\beta(a))\left\langle 1-\exp\left\{-\left[\lambda(t)+\sigma(a)+fp(t)\varepsilon(a)+\gamma(t)\right]\right\}\right\rangle q_j$

$+\frac{\varepsilon(a)bq(a)}{\left[\sigma(a)+\varepsilon(a)+n\alpha\right]}I_{01j}(t,a)\exp(-\mu(a))\left\langle 1-\exp\left\{-\left[\sigma(a)+\varepsilon(a)+n\alpha\right]\right\}\right\rangle$

(25) $I_{120j}(t+1,a+1)=I_{120j}(t,a)\exp\left\{-\left[\mu(a)+n\alpha\right]\right\}+$

$I_{120(j-1)}(t,a)\exp(-\mu(a))\left\langle 1-\exp\left\{-\left[+n\alpha\right]\right\}\right\rangle+$

$\frac{fp(t)\varepsilon(a)(1-q(a))}{\lambda(t)+\varepsilon(a)}S_{02}(t,a)\exp(-\mu(a))\left\langle 1-\exp\left\{-\left[\lambda(t)+\varepsilon(a)\right]\right\}\right\rangle q_j+$

$\frac{fp(t)\varepsilon(a)+\lambda(t)}{\lambda(t)+fp(t)\varepsilon(a)}S_{120}(t,a)\exp(-\mu(a))\left\langle 1-\exp\left\{-\left[\lambda(t)+fp(t)\varepsilon(a)\right]\right\}\right\rangle q_j+$

$\frac{\gamma(t)}{\gamma(t)+\rho(a)+n\alpha}I_{1010j}(t,a)\exp(-\mu(a))\left\langle 1-\exp\left\{-\left[\gamma(t)+\rho(a)+n\alpha\right]\right\}\right\rangle+$

$\frac{\gamma(t)+\sigma(a)}{\gamma(t)+\sigma(a)+n\alpha}I_{1110j}(t,a)\exp(-\mu(a))\left\langle 1-\exp\left\{-\left[\gamma(t)+\sigma(a)+n\alpha\right]\right\}\right\rangle$

$+\frac{\sigma(a)}{\left[\sigma(a)+n\alpha\right]}I_{1100j}(t,a)\exp(-\mu(a))\left\langle 1-\exp\left\{-\left[\sigma(a)+n\alpha\right]\right\}\right\rangle$

$+\frac{\varepsilon(a)(1-q(a))}{\left[\varepsilon(a)+n\alpha\right]}I_{02j}(t,a)\exp(-\mu(a))\left\langle 1-\exp\left\{-\left[\varepsilon(a)+n\alpha\right]\right\}\right\rangle$

$$(26)\ I_{121j}(t+1,a+1)=I_{121j}(t,a)\exp\left\{-\left[\mu(a)+\beta(a)+n\alpha\right]\right\}+$$
$$I_{121(j-1)}(t,a)\exp(-\mu(a)-\beta(a))\left\langle 1-\exp\left\{-\left[n\alpha\right]\right\}\right\rangle+$$
$$\frac{fp(t)\varepsilon(a)q(a)}{\lambda(t)+\varepsilon(a)}S_{02}(t,a)\exp(-\mu(a))\left\langle 1-\exp\left\{-\left[\lambda(t)+\varepsilon(a)\right]\right\}\right\rangle q_j+$$
$$\frac{fp(t)\varepsilon(a)+\lambda(t)}{\lambda(t)+fp(t)\varepsilon(a)}S_{121}(t,a)\exp(-\mu(a)-\beta(a))\left\langle 1-\exp\left\{-\left[\lambda(t)+fp(t)\varepsilon(a)\right]\right\}\right\rangle q_j+$$
$$\frac{\gamma(t)}{\gamma(t)+\rho(a)+n\alpha}I_{1011j}(t,a)\exp(-\mu(a)-\beta(a))\left\langle 1-\exp\left\{-\left[\gamma(t)+\rho(a)+n\alpha\right]\right\}\right\rangle+$$
$$\frac{\gamma(t)+\sigma(a)}{\gamma(t)+\sigma(a)+n\alpha}I_{1111j}(t,a)\exp(-\mu(a)-\beta(a))\left\langle 1-\exp\left\{-\left[\gamma(t)+\sigma(a)+n\alpha\right]\right\}\right\rangle$$
$$\frac{\sigma(a)}{\sigma(a)+n\alpha}I_{1101j}(t,a)\exp(-\mu(a)-\beta(a))\left\langle 1-\exp\left\{-\left[\sigma(a)+n\alpha\right]\right\}\right\rangle$$
$$+\frac{\varepsilon(a)q(a)}{\left[\varepsilon(a)+n\alpha\right]}I_{02j}(t,a)\exp(-\mu(a))\left\langle 1-\exp\left\{-\left[\varepsilon(a)+n\alpha\right]\right\}\right\rangle$$

In addition to the 26 variables we also calculate the cumulative number of individuals who have died due to the infection. These numbers we do not calculate for the individual ages separately, i.e. we sum all age groups. Since we have 13 types of infected individuals we define those who died in the corresponding 13 categories:

$$D_{00}(t+1)=D_{00}(t)+\sum_{a=1}^{100}\frac{n\alpha}{\rho(a)+\varepsilon(a)+n\alpha}I_{00n}(t,a)\exp(-\mu(a))\left\langle 1-\exp\left\{-\left[\rho(a)+\varepsilon(a)+n\alpha\right]\right\}\right\rangle+$$
$$q_{n+1}\sum_{a=1}^{100}\frac{\lambda(t)}{\lambda(t)+\varepsilon(a)+\rho(a)}S_{00}(t,a)\exp(-\mu(a))\left\langle 1-\exp\left\{-\left[\lambda(t)+\varepsilon(a)+\rho(a)\right]\right\}\right\rangle$$

$$D_{01}(t+1)=D_{01}(t)+\sum_{a=1}^{100}\frac{n\alpha}{\sigma(a)+\varepsilon(a)+n\alpha}I_{01n}(t,a)\exp(-\mu(a))\left\langle 1-\exp\left\{-\left[\sigma(a)+\varepsilon(a+n\alpha\right]\right\}\right\rangle+$$
$$q_{n+1}\sum_{a=1}^{100}\frac{\lambda(t)}{\lambda(t)+\varepsilon(a)+\sigma(a)}S_{01}(t,a)\exp(-\mu(a))\left\langle 1-\exp\left\{-\left[\lambda(t)+\varepsilon(a)+\sigma(a)\right]\right\}\right\rangle$$

$$D_{02}(t+1)=D_{02}(t)+\sum_{a=1}^{100}\frac{n\alpha}{\varepsilon(a)+n\alpha}I_{02n}(t,a)\exp(-\mu(a))\left\langle 1-\exp\left\{-\left[\varepsilon(a)+n\alpha\right]\right\}\right\rangle+$$
$$q_{n+1}\sum_{a=1}^{100}\frac{\lambda(t)}{\lambda(t)+\varepsilon(a)}S_{02}(t,a)\exp(-\mu(a))\left\langle 1-\exp\left\{-\left[\lambda(t)+\varepsilon(a)\right]\right\}\right\rangle$$

$$D_{1000}(t+1)=D_{1000}(t)+\sum_{a=1}^{100}\frac{n\alpha}{\rho(a)+n\alpha}I_{1000n}(t,a)\exp(-\mu(a))\left\langle 1-\exp\left\{-\left[\rho(a)+n\alpha\right]\right\}\right\rangle+$$
$$q_{n+1}\sum_{a=1}^{100}\frac{(1-b)fp(t)\varepsilon(a)(1-q(a))}{\lambda(t)+\varepsilon(a)+\rho(a)}S_{00}(t,a)\exp(-\mu(a))\left\langle 1-\exp\left\{-\left[\lambda(t)+\varepsilon(a)+\rho(a)\right]\right\}\right\rangle+$$
$$q_{n+1}\sum_{a=1}^{100}\frac{fp(t)\varepsilon(a)+\lambda(t)}{\lambda(t)+\varepsilon(a)+\rho(a)}S_{1000}(t,a)\exp(-\mu(a))\left\langle 1-\exp\left\{-\left[\lambda(t)+\varepsilon(a)+\rho(a)\right]\right\}\right\rangle$$

$$D_{1001}(t+1)=D_{1001}(t)+\sum_{a=1}^{100}\frac{n\alpha}{\rho(a)+n\alpha}I_{1001n}(t,a)\exp(-\mu(a)-\beta(a))\left\langle 1-\exp\left\{-\left[\rho(a)+n\alpha\right]\right\}\right\rangle+$$
$$q_{n+1}\sum_{a=1}^{100}\frac{(1-b)fp(t)\varepsilon(a)q(a)}{\lambda(t)+\varepsilon(a)+\rho(a)}S_{00}(t,a)\exp(-\mu(a))\left\langle 1-\exp\left\{-\left[\lambda(t)+\varepsilon(a)+\rho(a)\right]\right\}\right\rangle+$$
$$q_{n+1}\sum_{a=1}^{100}\frac{fp(t)\varepsilon(a)+\lambda(t)}{\lambda(t)+\varepsilon(a)+\rho(a)}S_{1001}(t,a)\exp(-\mu(a)-\beta(a))\left\langle 1-\exp\left\{-\left[\lambda(t)+\varepsilon(a)+\rho(a)\right]\right\}\right\rangle$$

$$D_{1010}(t+1)=D_{1010}(t)+\sum_{a=1}^{100}\frac{n\alpha}{\rho(a)+\gamma(t)+n\alpha}I_{1010n}(t,a)\exp(-\mu(a))\left\langle 1-\exp\left\{-\left[\rho(a)+\gamma(t)+n\alpha\right]\right\}\right\rangle+$$
$$q_{n+1}\sum_{a=1}^{100}\frac{bfp(t)\varepsilon(a)(1-q(a))}{\lambda(t)+\varepsilon(a)+\rho(a)}S_{00}(t,a)\exp(-\mu(a))\left\langle 1-\exp\left\{-\left[\lambda(t)+\varepsilon(a)+\rho(a)\right]\right\}\right\rangle+$$
$$q_{n+1}\sum_{a=1}^{100}\frac{fp(t)\varepsilon(a)+\lambda(t)}{\lambda(t)+fp(t)\varepsilon(a)+\rho(a)+\gamma(t)}S_{1010}(t,a)\exp(-\mu(a))\left\langle 1-\exp\left\{-\left[\lambda(t)+fp(t)\varepsilon(a)+\rho(a)+\gamma(t)\right]\right\}\right\rangle$$

$$D_{1011}(t+1)=D_{1011}(t)+\sum_{a=1}^{100}\frac{n\alpha}{\rho(a)+\gamma(t)+n\alpha}I_{1011n}(t,a)\exp(-\mu(a)-\beta(a))\left\langle 1-\exp\left\{-\left[\rho(a)+\gamma(t)+n\alpha\right]\right\}\right\rangle$$
$$+q_{n+1}\sum_{a=1}^{100}\frac{bfp(t)\varepsilon(a)q(a)}{\lambda(t)+\varepsilon(a)+\rho(a)}S_{00}(t,a)\exp(-\mu(a))\left\langle 1-\exp\left\{-\left[\lambda(t)+\varepsilon(a)+\rho(a)\right]\right\}\right\rangle+$$
$$q_{n+1}\sum_{a=1}^{100}\frac{fp(t)\varepsilon(a)+\lambda(t)}{\lambda(t)+fp(t)\varepsilon(a)+\rho(a)+\gamma(t)}S_{1011}(t,a)\exp(-\mu(a)-\beta(a))\left\langle 1-\exp\left\{-\left[\lambda(t)+fp(t)\varepsilon(a)+\rho(a)+\gamma(t)\right]\right\}\right\rangle$$

$$D_{1100}(t+1)=D_{1100}(t)+\sum_{a=1}^{100}\frac{n\alpha}{\sigma(a)+n\alpha}I_{1100n}(t,a)\exp(-\mu(a))\left\langle 1-\exp\left\{-\left[\sigma(a)+n\alpha\right]\right\}\right\rangle+$$
$$q_{n+1}\sum_{a=1}^{100}\frac{(1-b)fp(t)\varepsilon(a)(1-q(a))}{\lambda(t)+\varepsilon(a)+\sigma(a)}S_{01}(t,a)\exp(-\mu(a))\left\langle 1-\exp\left\{-\left[\lambda(t)+\varepsilon(a)+\sigma(a)\right]\right\}\right\rangle+$$
$$q_{n+1}\sum_{a=1}^{100}\frac{fp(t)\varepsilon(a)+\lambda(t)}{\lambda(t)+\varepsilon(a)+\sigma(a)}S_{1100}(t,a)\exp(-\mu(a))\left\langle 1-\exp\left\{-\left[\lambda(t)+\varepsilon(a)+\sigma(a)\right]\right\}\right\rangle$$

$$D_{1101}(t+1)=D_{1101}(t)+\sum_{a=1}^{100}\frac{n\alpha}{\sigma(a)+n\alpha}I_{1101n}(t,a)\exp(-\mu(a)-\beta(a))\left\langle 1-\exp\left\{-\left[\sigma(a)+n\alpha\right]\right\}\right\rangle+$$
$$q_{n+1}\sum_{a=1}^{100}\frac{(1-b)fp(t)\varepsilon(a)q(a)}{\lambda(t)+\varepsilon(a)+\sigma(a)}S_{01}(t,a)\exp(-\mu(a))\left\langle 1-\exp\left\{-\left[\lambda(t)+\varepsilon(a)+\sigma(a)\right]\right\}\right\rangle+$$
$$q_{n+1}\sum_{a=1}^{100}\frac{fp(t)\varepsilon(a)+\lambda(t)}{\lambda(t)+\varepsilon(a)+\sigma(a)}S_{1101}(t,a)\exp(-\mu(a)-\beta(a))\left\langle 1-\exp\left\{-\left[\lambda(t)+\varepsilon(a)+\sigma(a)\right]\right\}\right\rangle$$

$$D_{1110}(t+1)=D_{1110}(t)+\sum_{a=1}^{100}\frac{n\alpha}{\sigma(a)+\gamma(t)+n\alpha}I_{1110n}(t,a)\exp(-\mu(a))\langle 1-\exp\{-[\sigma(a)+\gamma(t)+n\alpha]\}\rangle+$$
$$q_{n+1}\sum_{a=1}^{100}\frac{bfp(t)\varepsilon(a)(1-q(a))}{\lambda(t)+\varepsilon(a)+\sigma(a)}S_{01}(t,a)\exp(-\mu(a))\langle 1-\exp\{-[\lambda(t)+\varepsilon(a)+\sigma(a)]\}\rangle+$$
$$q_{n+1}\sum_{a=1}^{100}\frac{fp(t)\varepsilon(a)+\lambda(t)}{\lambda(t)+\varepsilon(a)+\sigma(a)+\gamma(t)}S_{1110}(t,a)\exp(-\mu(a))\langle 1-\exp\{-[\lambda(t)+\varepsilon(a)+\sigma(a)+\gamma(t)]\}\rangle$$

$$D_{1111}(t+1)=D_{1111}(t)+\sum_{a=1}^{100}\frac{n\alpha}{\sigma(a)+\gamma(t)+n\alpha}I_{1111n}(t,a)\exp(-\mu(a)-\beta(a))\langle 1-\exp\{-[\sigma(a)+\gamma(t)+n\alpha]\}\rangle+$$
$$q_{n+1}\sum_{a=1}^{100}\frac{bfp(t)\varepsilon(a)q(a)}{\lambda(t)+\varepsilon(a)+\sigma(a)}S_{01}(t,a)\exp(-\mu(a))\langle 1-\exp\{-[\lambda(t)+\varepsilon(a)+\sigma(a)]\}\rangle+$$
$$q_{n+1}\sum_{a=1}^{100}\frac{fp(t)\varepsilon(a)+\lambda(t)}{\lambda(t)+\varepsilon(a)+\sigma(a)+\gamma(t)}S_{1111}(t,a)\exp(-\mu(a)-\beta(a))\langle 1-\exp\{-[\lambda(t)+\varepsilon(a)+\sigma(a)+\gamma(t)]\}\rangle$$

$$D_{120}(t+1)=D_{120}(t)+\sum_{a=1}^{100}I_{120n}(t,a)\exp(-\mu(a))\langle 1-\exp\{-[n\alpha]\}\rangle+$$
$$q_{n+1}\sum_{a=1}^{100}\frac{fp(t)\varepsilon(a)(1-q(a))}{\lambda(t)+\varepsilon(a)}S_{02}(t,a)\exp(-\mu(a))\langle 1-\exp\{-[\lambda(t)+\varepsilon(a)]\}\rangle+$$
$$q_{n+1}\sum_{a=1}^{100}\frac{p(t)\lambda(t)}{\lambda(t)+fp(t)\varepsilon(a)}S_{120}(t,a)\exp(-\mu(a))\langle 1-\exp\{-[\lambda(t)+fp(t)\varepsilon(a)]\}\rangle$$

$$D_{121}(t+1)=D_{121}(t)+\sum_{a=1}^{100}I_{121n}(t,a)\exp(-\mu(a)-\beta(a))\langle 1-\exp\{-[n\alpha]\}\rangle+$$
$$q_{n+1}\sum_{a=1}^{100}\frac{fp(t)\varepsilon(a)q(a)}{\lambda(t)+\varepsilon(a)}S_{02}(t,a)\exp(-\mu(a))\langle 1-\exp\{-[\lambda(t)+\varepsilon(a)]\}\rangle+$$
$$q_{n+1}\sum_{a=1}^{100}\frac{p(t)\lambda(t)}{\lambda(t)+fp(t)\varepsilon(a)}S_{121}(t,a)\exp(-\mu(a)-\beta(a))\langle 1-\exp\{-[\lambda(t)+fp(t)\varepsilon(a)]\}\rangle$$

## References (Appendix)

1. Mode CJ. Stochastic processes in demography and their computer implementation. Berlin ; New York: Springer-Verlag; 1985. xvii, 389 p. p. (Biomathematics ; v. 14).
2. Thiele PN. On a mathematical formula to express the rate of mortality throughout the whole life. J Inst Actuaries. 1872; 16:213-39.
3. Wallis JP, Wells AW, Matthews JN, Chapman CE. Long-term survival after blood transfusion: a population based study in the North of England. Transfusion. 2004; 44:1025-32.
